# Metal-based nanomedicines for cancer theranostics

**DOI:** 10.1186/s40779-025-00627-x

**Published:** 2025-07-30

**Authors:** Hai-Jia Yu, Jian-Hua Liu, Wei Liu, Rui Niu, Bin Zhang, Yuan Xiong, Yang Liu, Ying-Hui Wang, Hong-Jie Zhang

**Affiliations:** 1 Department of Radiology, The Second Hospital of Jilin University, Changchun, 130041 China; 2 State Key Laboratory of Rare Earth Resource Utilization, Changchun Institute of Applied Chemistry, Chinese Academy of Sciences, Changchun, 130022 China; 3 Department of Chemistry, Tsinghua University, Beijing, 100084 China; 4 School of Applied Chemistry and Engineering, University of Science and Technology of China, Hefei, 230026 China; 5 Department of Orthopedics, Tongji Hospital, Tongji Medical College, Huazhong University of Science and Technology, Wuhan, 430030 China; 6 School of Chemistry, Chemical Engineering and Biotechnology, Nanyang Technological University, Singapore, 637371 Singapore

**Keywords:** Metal-based nanomedicines, Synthesis strategy, Computed tomography imaging, Nuclear imaging, Magnetic resonance imaging, Fluorescence imaging, Photoacoustic imaging, Drug-delivery, Phototherapy, Catalytic therapy, Ion interference therapy, Immunotherapy

## Abstract

The heterogeneity and invasiveness of cancer cells pose serious challenges in cancer diagnosis and treatment. Advancements and innovations in metal-based nanomedicines provide novel avenues for addressing these challenges. Metal-based nanomedicines possess unique physicochemical properties that enable their interaction with living organisms, thereby inducing complex biological responses. These nanomaterials have been extensively used to enhance the contrast and sensitivity of cancer imaging and to amplify the distinction between cancerous and healthy tissues. Moreover, these nanomaterials can effectively combat a wide spectrum of cancers through various methods, including drug delivery, radiotherapy, photothermal therapy (PTT), photodynamic therapy (PDT), sonodynamic therapy (SDT), biocatalytic therapy, ion interference therapy (IIT), and immunotherapy. Currently, there is still a need for a comprehensive summary on the metal-based nanomaterials for cancer diagnosis and treatment. Herein, we present a systematic and complete overview of action mechanisms and the applications of metal-based nanomaterials in cancer theranostics. A summary of common strategies for synthesizing and modifying metal-based nanomedicines is presented, and their biosafety is analyzed. Then, the latest developments in their applications for cancer imaging and anticancer treatment are provided. Finally, the key technical challenges and reasonable perspectives of metal-based nanomedicines for cancer theranostics in clinical applications are discussed.

## Background

Cancer remains one of the most serious threats to human health worldwide, with annual increases in morbidity and mortality rates [1]. In 2024, there were estimated to be 3,246,625 and 2,510,597 new cancer cases, along with 1,699,066 and 640,038 cancer deaths in China and the USA, respectively [2]. It is anticipated that approximately 28.4 million individuals will be diagnosed with cancer worldwide by 2040 [3]. Currently, although imaging approaches such as ultrasound (US), computed tomography (CT), and magnetic resonance imaging (MRI) have offered a macroscopic view and anatomical information for cancer detection, the identification of subtle abnormalities before evident anatomical changes remains a challenge [4, 5]. Conventional cancer treatments, such as surgery, chemotherapy, and radiotherapy, have achieved a certain level of success in terms of decreasing tumor size and inhibiting tumor growth in vivo. However, due to the indiscriminate attack on both tumor and normal tissues, coupled with tumor multidrug resistance, the efficacy of traditional approaches often proves inadequate, leading to a high recurrence and metastasis rate, as well as obvious toxic side effects, which in turn increase the difficulty of tumor treatment [6, 7]. Therefore, developing novel methods for sensitive and accurate cancer detection, as well as achieving complete eradication with minimal side effects, is crucial to improving the survival prognosis of individuals with cancer, which represents both a challenge and an opportunity for precise diagnosis and anticancer therapy.

Metal-based nanomaterials, including metal-based anticancer drugs (small molecules), pure metal nanomaterials, metal alloy nanomaterials, metal compounds [metal oxides, fluorides, phosphides, carbides, nitrides, borides, chlorides, chalcogenides, layered double hydroxides (LDHs)], metal nanoclusters, metal nanocrystals, metal complexes, metal–organic frameworks (MOFs), and metal-doped covalent organic frameworks (COFs), possess distinct inherent optical, magnetic, thermal, and electronic properties, which hold great promise for early cancer diagnosis and optimization of therapeutic efficacy [8–11]. It is possible to regulate the biodistribution and circulation time of metal-based nanomaterials in vivo by altering fundamental parameters such as size, surface charge, and geometry [12, 13]. Moreover, based on the characteristics of metal-based nanomaterials, including large surface area, high fraction surface atoms, and high surface reactivity, specific targeting groups and organic components can be readily modified on their surface to improve stability, enhance biocompatibility, and tailor multifunctional nanoimaging or nanotherapeutic agents with tumor-targeting ability, high permeability, and stimuli responsiveness [14, 15].

Benefiting from the unique advantages of metal-based nanomaterials, remarkable progress has been made in efficient cancer imaging and treatment, especially in the following aspects: 1) through surface modification, metal-based nanomaterials can specifically bind to targets overexpressed in cancer tissues in vivo, which allows for the detection of subtle changes in cancer occurrence by tracking the dynamic distribution and accumulation of these nanomaterials using imaging techniques, thus enabling personalized cancer diagnosis at an early stage [16]; 2) the integration of different imaging modalities into a metal-based nanosystem enables the provision of additional information for detecting primary cancers and distant metastases [17]; 3) the utilization of metal-based nanomaterials as carriers for therapeutic drugs facilitates targeted delivery and controlled release, thereby increasing the drug concentration at tumor sites to optimize therapeutic efficacy while limiting a wide distribution in healthy cells and mitigating systemic side effects [7]; 4) metal-based nanomaterials with high atomic numbers have been demonstrated to sensitize radiotherapy through several pathways, including increasing radiation energy deposition, enhancing reactive oxygen species (ROS) production, inducing cell cycle arrest in sensitive G2/M phases, intensifying DNA damage, and inhibiting DNA damage repair [18]; 5) some metal-based nanomaterials have been proven to exhibit properties such as localized surface plasmon resonance (LSPR), excellent near-infrared (NIR) light absorption, and high photothermal conversion efficiency (PCE), which help convert NIR laser irradiation into heat for effective cancer ablation [19]; 6) metal-based nanomaterials can transport photosensitizers (PSs) and sonosensitizers to specific tumor regions or act as PSs and sonosensitizers themselves to realize anticancer photodynamic therapy (PDT) and sonodynamic therapy (SDT) [20, 21]; 7) some metal-based nanomaterials have shown redox abilities and exhibit Fenton/Fenton-like and enzyme-mimicking catalytic activities, which contribute to achieving cancer eradication through biocatalytic therapy [22]; 8) metal-based nanomaterials are reported to increase the intracellular concentration of metal ions, which can effectively inhibit cancer growth by interfering with osmolality (Na^+^, K^+^, and Ba^2+^), affecting signal transduction (Ca^2+^ and Zn^2+^), and damaging deoxyribonucleic acid (DNA) (Pt^2+^ and Ag^+^) [23]; and 9) metal-based nanomaterials have been demonstrated to modulate the immunosuppressive tumor microenvironment (TME) and enhance antigen presentation by delivering immunomodulatory substances to specific targets, inducing immunogenic cell death (ICD), or acting as potent immune adjuvants, thereby realizing anticancer immunotherapy [24]. As a result, innovative strategies based on metal-based nanomaterials will pave the way for safer and more effective cancer diagnosis and treatment.

**Fig. 1:**
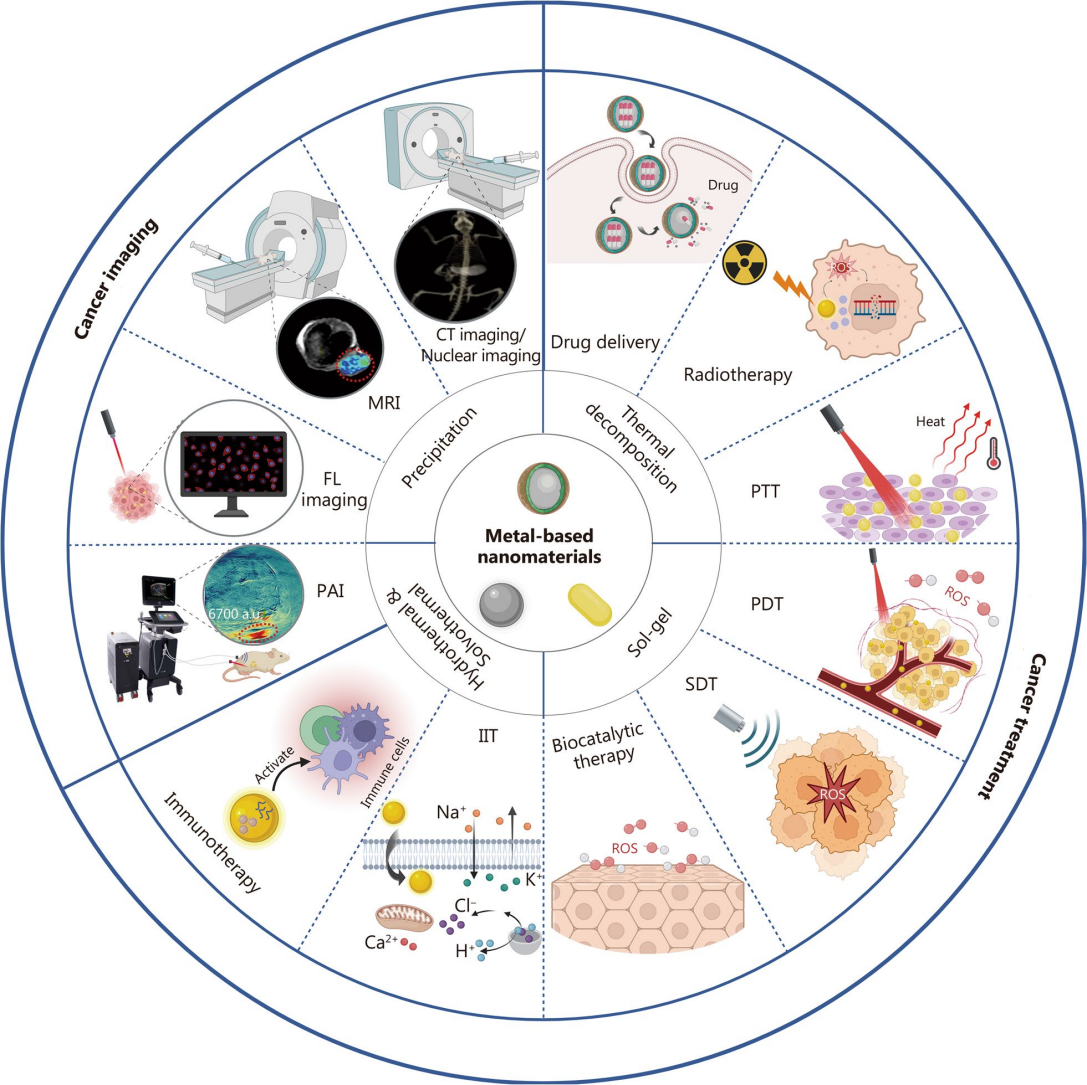
Overview of metal-based nanomedicines for cancer theranostics. The commonly used methods for synthesizing metal-based nanomaterials include precipitation, hydrothermal synthesis, solvothermal synthesis, thermal decomposition, and the sol-gel method. In cancer imaging applications, metal-based nanomaterials are widely employed in CT imaging, nuclear imaging, MRI, FL imaging, and PAI. For cancer treatment, these nanomaterials exhibit therapeutic potentials via diverse modalities, including functioning as DDS for targeted agent transport, facilitating radiotherapy, PTT, PDT, SDT, catalytic therapy, IIT, and immunotherapy. It was created in BioRender. CT computed tomography, MRI magnetic resonance imaging, FL fluorescence, PAI photoacoustic imaging, PTT photothermal therapy, PDT photodynamic therapy, SDT sonodynamic therapy, IIT ion interference therapy

**Fig. 2:**
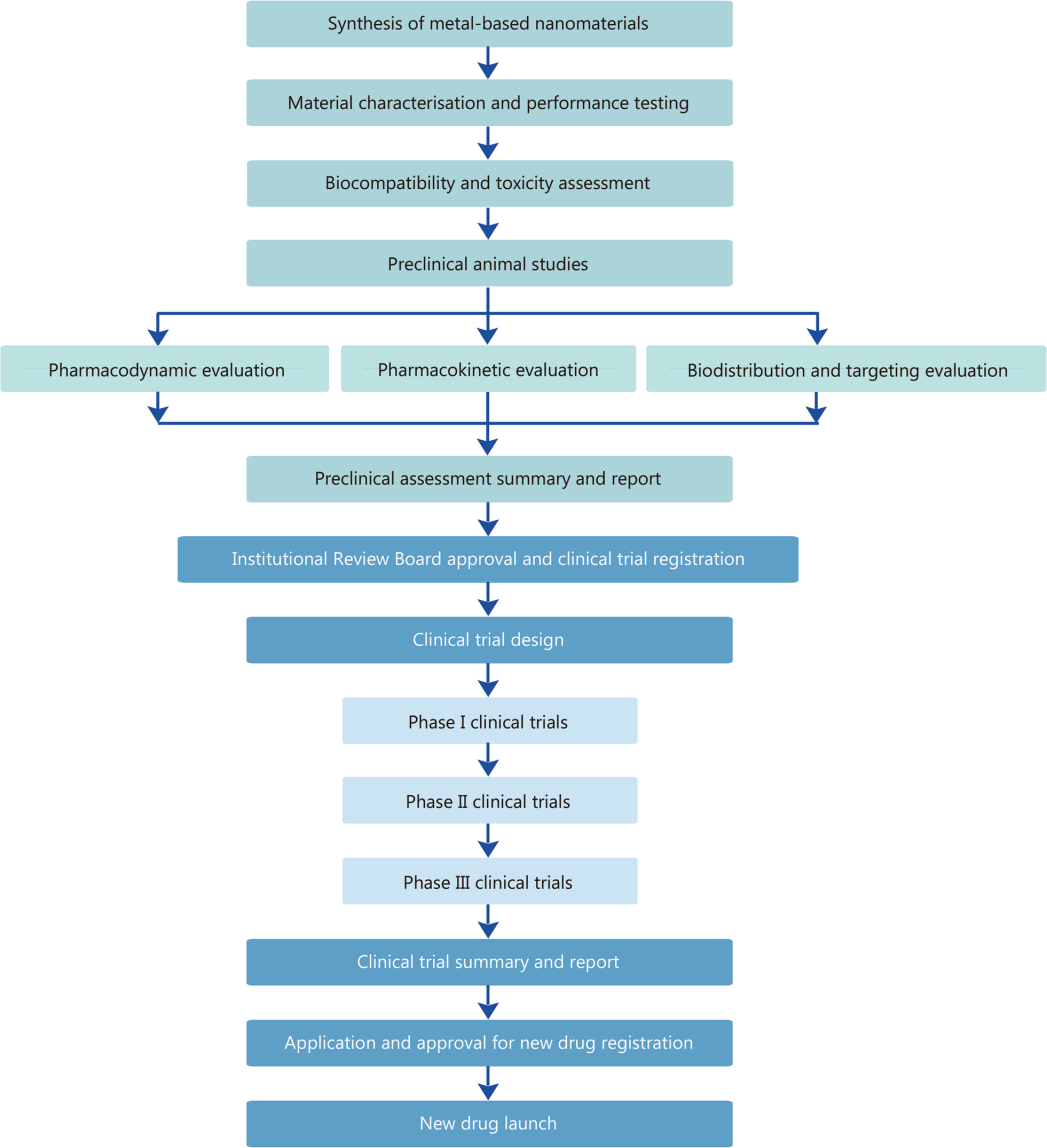
Flowchart of the entire process of developing metal-based nanomaterials as agents for tumor theranostics. The process begins with the synthesis of metal-based nanomaterials, progresses through performance and toxicity evaluation, preclinical animal studies, clinical trials, regulatory approval for new drug registration, and ultimately reaches the launch of new nanodrugs

Although metal-based nanomaterials have been reviewed for cancer theranostics [25, 26], there is still a pressing need for a more comprehensive summary of this field. In this review, we present a more systematic and comprehensive overview of the mechanisms and applications of metal-based nanomaterials in cancer theranostics. We summarize the general strategies for synthesizing and modifying metal-based nanomaterials and discuss their biosafety. Then, we provide an overview of the role of metal-based nanomaterials in cancer imaging, including CT imaging, nuclear imaging, MRI, fluorescence (FL) imaging, photoacoustic imaging (PAI), and multimodal imaging. Thereafter, we focus on the applications of metal-based nanomaterials in anticancer treatment, including drug delivery, radiotherapy, photothermal therapy (PTT), PDT, SDT, biocatalytic therapy, ion interference therapy (IIT), immunotherapy, and multimodal therapy (Fig. 1). The metal-based nanomaterials used for tumor imaging and treatment are summarized in Table 1. Figure 2 describes the entire process of developing metal-based nanomaterials as agents for tumor theranostics. This review concludes with a discussion of the key technical challenges and prospects of metal-based nanomaterials in cancer applications.

**Table 1:** Summary of metal-based nanomaterials for tumor imaging and treatment

Materials	Imaging	Treatment
GNPs-PEG@cNGR	CT	–
PEGylated Au nanomaterials	CT	–
BiOCl nanosheets	CT	–
PEG-Bi nanomaterials	CT	–
BMSN-AR nanomaterials	CT	–
PAA-coated metal oxide (Yb_2_O_3_, NaTaO_3_, Dy_2_O_3_, and Gd_2_O_3_) nanomaterials	CT	–
MoWO nanobundles	CT	–
^99m^Tc-doped IO-PVP	SPECT	–
^64^Cu-CuNCs-FC131	PET	–
PEG-Gd_2_O_3_ nanomaterials	T1-weighted MRI	–
Gd-SA	T1-weighted MRI	–
L-EGCG-Mn nanomaterials	T1-weighted MRI	–
Mn@CCs	T1-weighted MRI	–
MnO nanomaterials	T1-weighted MRI	–
Anchor-free PEGylated Fe_3_O_4_ nanomaterials	T2-weighted MRI	–
FA@Fe_3_O_4_ nanomaterials	T2-weighted MRI	–
Fe_3_O_4_-PEG-DG nanomaterials	T2-weighted MRI	–
PEG-PATU Ag_2_S QDs	NIR-II FL imaging	–
CISe@ZnS QDs	NIR-II FL imaging	–
NaYF_4_:Yb,Er,Eu@NaYF_4_:Nd nanomaterials	UCL/NIR-II FL imaging	–
CSS@P-PAA	NIR-II FL imaging	PTT/PDT
GNRs@Chit-Iso4	PAI	–
GNRs@PEG-Iso4	PAI	–
GNR@SiO_2_@MnO_2_	MRI/PAI	–
DNA-Ag@Pd nanoclusters	PAI	PTT
HASAIC	CT/PAI	PTT/SDT
AuNCs-LHRHa nanosystem	CT/FL imaging	PTT
AuNCs-A@PAA/CaP	CT/FL imaging	Chemotherapy
FeGdNP-ICG/GOx-RGD2-mPEG	MRI/FL imaging	Ferroptosis
CuS/Gd_2_O_3_ nanomaterials	MRI/FL imaging	–
NaGdF_4_-PEG-cMBP	MRI/FL imaging	–
Au/Gd@FA nanomaterials	MRI/FL imaging	–
SiO_2_@Ag nanoprobes	FL/PAI	–
AMNDs-LHRH	FL/CT/MRI	–
Bi/MnPcE_4_ nanocomposites	FL/CT/MRI	PDT
NaYbF_4_:Tm^3+^/Gd^3+^ nanorods	UCL/CT/MRI	–
Bi-QCS-AuNPs@collagen	–	Drug delivery
CuS-MoS_2_-SH-PEG(DOX)	–	Drug delivery/PTT/Chemotherapy
MOF nanoswitch	–	Drug delivery
FA-PEG-Pam/CaP/NDs	–	Drug delivery
Chitosan-coated CuO nanomaterials	–	Drug delivery
NP-3pRNA-CpG	–	Drug delivery
IO@FuDex^3^	–	Drug delivery
Au nanostructures	–	Radiotherapy
IO@Ag nanomaterials	–	Radiotherapy
Hollow PtCo nanospheres	–	Radiotherapy
M/H–D	CT/PAI	PTT/Radiotherapy/Catalytic therapy
HfO_2_@Au core–shell nanomaterials	–	Radiotherapy
Hensify®	–	Radiotherapy
AGuIX	MRI	Radiotherapy
MnCO-Tw-SCNPs	–	Radiotherapy
AuNR@BSA	–	PTT
DMSN-Au-Ru	–	PTT/Radiotherapy/Catalytic therapy
Bi_19_S_27_I_3_ nanorods	–	PTT
CuS@Cu-MOF	–	Drug delivery/PTT/CDT
Au@mSiO_2_-ICG	–	PTT/PDT
AlPcS_4_Cl-AuNP-Ab	–	PDT
IONC-PEG-Ce6	MRI/FL imaging	PDT
MOF@MOF nanoplatforms	–	PDT
Au nanosheets	–	PDT
BSA-Ag_13_ nanoclusters	–	PDT
H-Cu_9_S_8_@CCM nanomaterials	–	PTT/SDT
GNP-protoporphyrin IX conjugate	–	SDT
Zr-MOF@AIPH	–	SDT
Au/TiO_2_ nanocomposites	–	PTT/SDT
Au-TiO_2_ nanomaterials	–	SDT
D-ZnO_x_:Gd	–	SDT
D-MOF(Ti)	–	SDT
PMCS	–	SDT
Metal-porphyrin complexes (ZnTTP, MnTTP, and TiOTTP)	–	SDT
FP nanomaterials	MRI/PAI	PTT/CDT
CP nanomaterials	MRI	PTT/CDT
GA-Fe@HMDN-PEI-PEG	–	CDT
MnVO_3_ nanomaterials	–	CDT
CoS_x_ QDs	–	PTT/CDT
CMO nanomaterials	–	PTT/PDT/CDT
AuCuPt-protoporphyrin IX nanozyme	–	SDT/Catalytic therapy
FePOs nanozyme	–	Catalytic therapy
Ir–N_5_ SAzyme	–	Catalytic therapy
CHO-loaded Co–PN_3_ SAzyme	–	Catalytic therapy
PNSO nanomaterials	–	IIT/Immunotherapy
GL-BaO_2_ nanomaterials	–	IIT
SH-CaO_2_ nanomaterials	–	IIT
MnO_2_-Pt@Au_25_ nanosheets	–	PDT/IIT/Chemotherapy
FTP	–	Catalytic therapy/Immunotherapy
TiO_2_@CaP nanoagents	–	SDT/Immunotherapy
CoFe_2_O_4_@MnFe_2_O_4_ nanomaterials	–	Magnetic hyperthermia therapy/Immunotherapy
PL/APMP-DOX nanomaterials	–	Chemotherapy/Immunotherapy
Zn-LDH	–	Immunotherapy
CaCO_3_ nanomaterials	–	Immunotherapy
M-Pt/PEG-CuS	–	PTT/Chemotherapy
CDAuNs	–	PTT/Chemotherapy
MoSe_2_@ICG-PDA-HA	–	PTT/PDT
CFNs	–	PTT/PDT/CDT
Bi_2_S_3−x_-Au@HA heterostructure nanocomposites	–	PTT/PDT/SDT

*GNPs* gold nanoparticles, *PEG* polyethylene glycol, *GNPs-PEG@cNGR* GNPs modified with PEG and a cyclized asparagine-glycine-arginine peptide, *CT* computed tomography, *BMSN* Bi-based mesoporous-silica-coated nanomaterial, *AR* MCF-7 tumor-targeted peptide, *PAA* polyacrylic acid, *SPECT* single-photon emission computed tomography, *IO* iron oxide, *PVP* polyvinylpyrrolidone, *NC* nanocluster, *PET* positron emission tomography, *MRI* magnetic resonance imaging, *Gd-SA* single-atom Gd nanocontrast agent, *L-EGCG* L-epigallocatechin gallate, *Mn@CCs* carbonized complexes of Mn^2+^, *FA* folic acid, *DG* D-glucosamine, *PATU* polyacylthiourea dendrimer, *QDs* quantum dots, *NIR* near-infrared, *FL* fluorescence, *UCL* upconversion luminescence, *CSS* NaYF_4_:Yb^3+^/Er^3+^@NaYF_4_:Nd^3+^@NaYF_4_, *P* PFC-55 hydrogen-bonded organic frameworks, *PTT* photothermal therapy, *PDT* photodynamic therapy, *GNRs* gold nanorods, *Chit* chitosan, *PAI* photoacoustic imaging, *DNA* deoxyribonucleic acid, *HASAIC* hollow Ag_2_S/Ag@I/chlorin e6, *SDT* sonodynamic therapy, *LHRHa* luteinizing hormone-releasing hormone analogues, *A* assemblies, *ICG* indocyanine green, *GOx* glucose oxidase, *RGD2* RGD dimer, *cMBP* cMet-binding peptide, *AMNDs* Au/Mn nanodots, *MnPcE*_*4*_ manganese phthalocyanine substituted with a carboxyl, *Bi-QCS* biotin-quat188-chitosan, *DOX* doxorubicin, *MOF* metal–organic framework, *Pam* pamidronate, *CaP/NDs* CaP/nuclear localization signal/plasmid DNA, *NP-3pRNA-CpG* nano sized aluminum hydroxide combined cytosine-phosphate-guanine oligodeoxynucleotide and 5′-triphosphate RNA, *Fu* fucoidan, *Dex* aldehyde-functionalized dextran, *M/H–D* MoS_2_/HfO_2_ dextran, *Tw* Tween-20, *SCNPs* scintillating nanoparticles, *BSA* bovine serum albumin, *DMSN* dendritic mesoporous silica, *CDT* chemodynamic therapy, *AlPcS*_*4*_*Cl* Al(III) phthalocyanine chloride tetra sulfonic acid, *Ab* antibody, *Ce6* chlorin e6, *H* hemoporfin, *CCM* CT26 cell membrane, *AIPH* 2,2-azobis[2-(2-imidazolin-2-yl)propane] dihydrochloride, *D-ZnO*_*x*_*:Gd* defect-rich Gd doped ZnO, *D-MOF(Ti)* defect-rich Ti-based MOF, *PMCS* MOF-derived carbon nanostructure that contains porphyrin-like metal centers, *TTP* 4-methylphenylporphyrin, *FP* ferrous phosphide, *CP* copper(I) phosphide, *GA-Fe* gallic acid-ferrous, *HMDN* hollow mesoporous MnO_2_ nanoparticle, *PEI* polyethyleneimine, *CMO* Ce-doped MoO_x_*, SAzyme* single-atom nanozyme, *CHO* cholesterol oxidase, *PNSO* phospholipid-modified Na_2_S_2_O_8,_ *GL-BaO*_*2*_ BaO_2_ nanomaterial coated with the chelator *N,N*-bis(carboxymethyl)-_L_-glutamic acid tetrasodium salt, *SH* sodium-hyaluronate, *FTP* Fe-protoporphyrin-based hybrid metal–organic frameworks, *PL* phospholipid, *APMP* amorphous porous manganese phosphate, *LDH* layered double hydroxide, *CDAuNs* gold nanocages coated with 4T1 cancer cell membranes and loaded with DOX, *PDA* polydopamine, *HA* hyaluronic acid, *CFNs* copper ferrite nanospheres

## Synthesis, modification, and biosafety of metal-based nanomaterials

### Synthesis strategies for metal-based nanomaterials

**Fig. 3:**
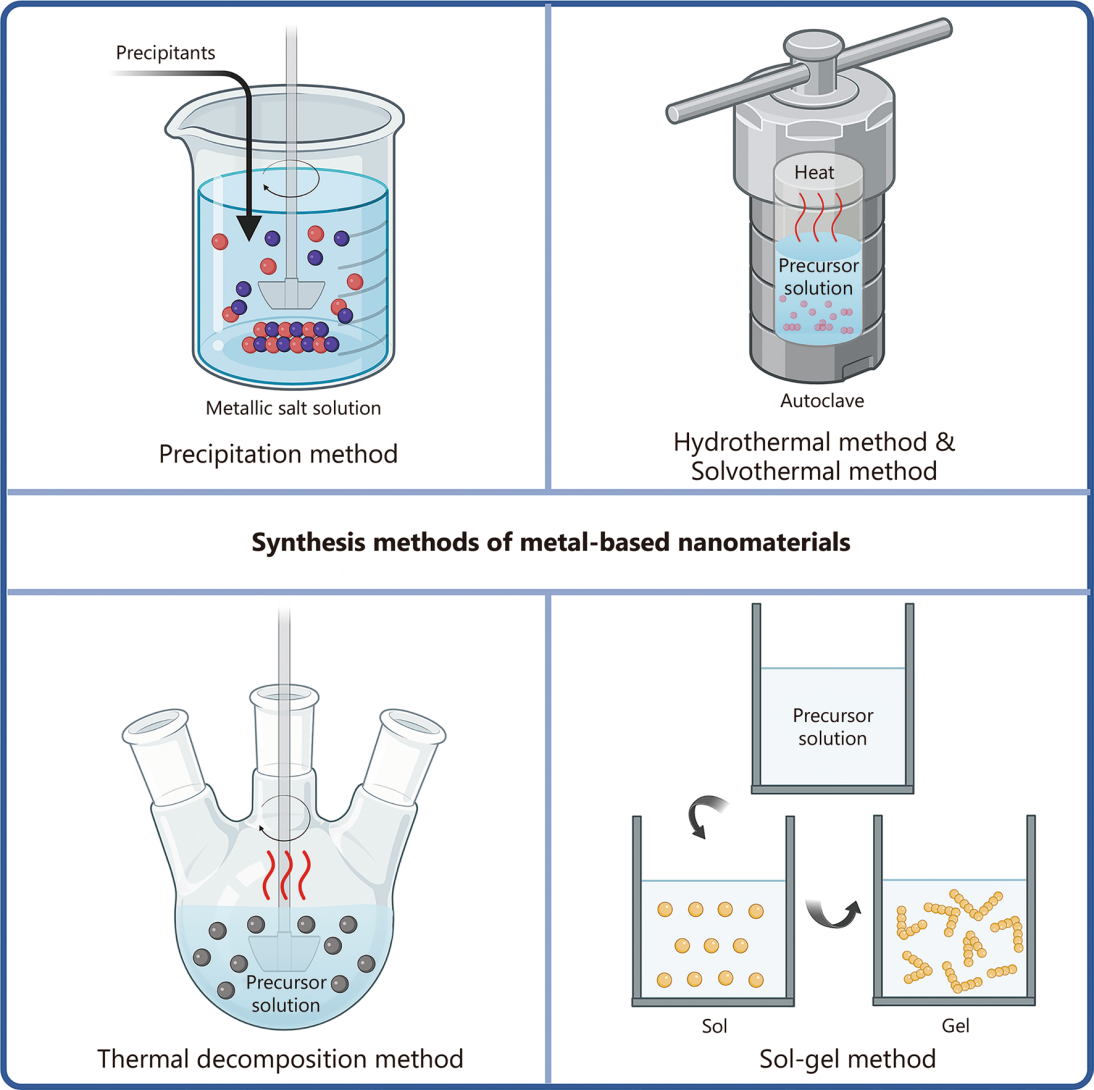
Schematic of synthesis methods for metal-based nanomaterials. The common methods for synthesizing these materials include precipitation, hydrothermal synthesis, solvothermal synthesis, thermal decomposition, and the sol-gel method. It was created in BioRender

To date, various metal-based nanomaterials with distinct structures, sizes, morphologies, and chemical compositions have been designed for use in nanomedicine. These fundamental parameters, which are closely related to the physicochemical properties of metal-based nanomaterials, have laid the foundation for the use of metal-based nanomaterials as cancer imaging or therapeutic agents. As the size decreases, metal-based nanomaterials tend to exhibit an increased ability to extravasate from tumor blood vessels and penetrate deeper into tumor tissues [27]. Rod-shaped metal-based nanomaterials are more likely to accumulate in the tumor parenchyma compared to their spherical or other geometrically shaped counterparts [28]. Therefore, appropriate synthesis strategies should be selected with precise control over reaction conditions to modulate the basic parameters of metal-based nanomaterials and meet the requirements for cancer diagnosis and treatment. In general, the main strategies for synthesizing metal-based nanomaterials can be divided into two categories: physical and chemical methods. Physical preparation approaches, such as grinding and sputtering, are simple to operate and are commonly used for synthesizing metal-based nanomaterials [29, 30]. Nevertheless, the undesired shape and size of the obtained nanomaterials restrict their direct in vivo applications. In contrast, metal-based nanomaterials prepared by chemical methods have the advantages of uniform size distribution, controllable morphology, and facile surface functionalization. Conventional chemical preparation methods include precipitation, hydrothermal synthesis [31], solvothermal synthesis [32], thermal decomposition [33], and the sol-gel method [34] (Fig. 3). Here, we introduce the mechanisms and several typical examples of these methods. The advantages and disadvantages of metal-based nanomaterial synthesis strategies are summarized in Table 2.

**Table 2:** Summary of the advantages and disadvantages of synthesis strategies for metal-based nanomaterials

Synthesis strategies	Advantages	Disadvantages
Precipitation method	Simple process, convenient operation, rapid reaction, low cost, easy to industrialize	Difficult to control crystal shape and separate products, low product purity
Hydrothermal method	Facile operation, high product purity, high crystallinity of nanocrystals, low melting point, low energy consumption, controllable product shape and size, low pollution levels	Inability to directly observe crystal growth, expensive autoclaves, long production time
Solvothermal method	High product purity, good product dispersion, controllable nanostructure morphology	Unobservable synthesis process, high cost, low product yield, potential safety hazards
Thermal decomposition method	High product purity, controllable product shape and size, narrow product size distribution	High equipment cost, high energy consumption, complex operation, small output
Sol-gel method	Low cost, high product purity, controllable product shape and size	Long preparation time, unstable product structure, product is prone to agglomeration

#### Precipitation method

The precipitation method typically involves the addition of a precipitant to a mixture of solutions containing different chemicals. Hydroxides [35], carbonates [36], and oxalates [37, 38] are typical precipitants. When the concentration product of ions in a solution exceeds the solubility product, cations and anions bind together to form crystal nuclei [39]. Subsequently, the crystal size increases as the solute continuously deposits, leading to the formation of an insoluble precursor precipitate [39]. On this basis, impurities mixed in the precursor products can be removed through processes such as filtration and washing. Ultimately, metal-based nanomaterials with the desired structures, morphologies, and dimensions can be obtained through processes such as drying or calcination [40, 41]. For example, Liu et al. [42] successfully synthesized hollow MnO_2_ nanoshells via the redox deposition method for efficient loading of chemotherapeutic drugs, thereby facilitating enhanced treatment of colorectal cancer. Moreover, Zhang et al. [43] prepared CaO_2_ nanomaterials by adding H_2_O_2_ to a mixed solution containing ammonia, CaCl_2_, and polyethylene glycol (PEG), followed by adjusting the pH to 11.5 using NaOH. Notably, the use of capping agents or surfactants is beneficial to synthesize metal-based nanomaterials with a controlled particle size and uniform distribution. Specifically, during the crystal growth phase, agglomeration or Ostwald ripening is likely to occur to reduce surface energy, which may lead to an excessive increase in the size of metal-based nanomaterials. In this case, by adding capping agents or surfactants, the electrostatic repulsion between metal-based nanomaterials can be enhanced, thereby achieving steric stabilization and preventing coagulation [44]. For instance, Bleier et al. [45] employed alkylated pentanediones as surfactants to reduce magnetic nanomaterial agglomeration.

Co-precipitation has been recognized as a facile approach for the fabrication of metal-based nanomaterials. By introducing a precipitant into a homogeneous metallic salt solution prepared in specific proportions, the co-precipitation method enables the complete precipitation of all metal cations from the solution. In this way, various metal-based nanostructures such as pure metal nanomaterials, metal alloy nanomaterials, metal compounds, MOFs, and metal complexes with uniform size distributions can be easily obtained [46–48]. For example, Darwish et al. [49] used NH_4_OH as a precipitant and added it to a solution containing Fe^3+^ and Fe^2+^ to obtain precursor precipitates at 70°C. Subsequently, polyvinyl alcohol was introduced as a surfactant to fabricate monodisperse, stable magnetite nanomaterials with good hyperthermia properties. Similarly, Chin et al. [50] synthesized highly dispersed Fe_3_O_4_@Chl/Fe nanoclusters using the co-precipitation method by adding NH_4_OH to a solution containing FeCl_3_, FeCl_2_, and iron chlorophyll at room temperature.

#### Hydrothermal method

Hydrothermal synthesis is a widely employed approach for fabricating diverse metal-based nanomaterials, such as metal nanomaterials, metal alloy nanomaterials, metal compounds, MOFs, and metal-based COFs [51–55]. The hydrothermal process is conducted in a sealed autoclave at high temperatures, where metal precursors are used as raw materials, and supercritical water is used as the solvent. The metal-based nanomaterials are characterized by their small size and narrow size distribution. Mineralizers are often introduced into the reaction system to further enhance the solubility of the precursor. Certain reaction parameters, including the reaction time, temperature, pH, and precursor concentration, affect the structure, size, and morphology of nanomaterials [31, 56]. Furthermore, surface modification is an alternative approach to effectively modulate the morphology of metal-based nanomaterials [57]. In one typical example, Liu et al. [31] investigated the influence of different reaction conditions on the physical properties of ZrO_2_ nanomaterials by altering process variables during hydrothermal synthesis. As a result, the size of ZrO_2_ nanomaterials exhibited a steady increase with prolonged reaction time or increased precursor concentration, while altering the pH value of the reaction system resulted in diverse crystal structures.

Compared to other chemical preparation methods, hydrothermal treatment has the advantages of facile operation, rapid reaction, and high product purity, which have attracted considerable attention among researchers. For example, Wang et al. [58] successfully prepared iron-based nanosheets by a hydrothermal method using sodium salicylate, NaOH, and Fe(NO_3_)_3_ in an autoclave at 120°C. They found that these nanosheets exhibited high purity and excellent photothermal conversion performance, as well as drug-loading capability. In another study, PEG-coated yttrium-doped ZnO nanomaterials with a narrow size distribution were conveniently synthesized via a low-cost one-step hydrothermal process, which displayed photoluminescence and X-ray attenuation properties, along with PDT performance [59]. Homogeneous and stable LDH nanomaterials can be synthesized with high repeatability through co-precipitation and hydrothermal synthesis. For example, Xu et al. [60] precipitated MgAl-LDH by introducing a mixed solution containing MgCl_2_ and AlCl_3_ into NaOH solution via the co-precipitation method. Subsequently, stable and homogeneous Mg_2_Al-Cl-LDH suspensions were successfully synthesized via a hydrothermal method in an autoclave at 100°C using the MgAl-LDH solution. This facile and reproducible synthesis method provides a new approach for the large-scale production of LDH nanomaterials.

#### Solvothermal method

Based on the hydrothermal process, the solvothermal method employs organic solvents, such as ethanol, ethylene glycol, and butanediol, instead of water as the reaction medium to realize the controllable synthesis of metal-based nanomaterials with various structures, sizes, and morphologies by adjusting the composition and amount of solvents [61–64]. For example, Asakura et al. [65] synthesized a range of metal oxyfluorides with diverse morphologies through solvothermal reactions of precursors (MoO_3_ and WO_3_) with NaF in different organic solvent systems. The products synthesized using acetonitrile as the solvent exhibited a rod-like shape, whereas those prepared using ethanol as the solvent displayed a polyhedral shape. Differences in the solubility of reactants in different organic solvents can also affect particle size. The solvothermal reactions of MoO_3_, WO_3_, and NaF in organic solvents with higher solubility facilitated the synthesis of smaller particles.

In addition to altering the solvent type, suitable organic ligands are commonly used to regulate the size and morphology of solvothermal products [66]. In one representative example, Duong et al. [66, 67] utilized oleic acid as a surfactant to prepare a series of cobalt iron oxide nanomaterials through the solvothermal method. Compared to the bare large-sized cubic nanomaterials, the oleic acid-coated nanomaterials were spherical, with smaller sizes and a more homogeneous size distribution. The observed outcomes could be attributed to the improved stabilization of nucleation and crystal growth by oleic acid.

#### Thermal decomposition method

The typical thermal decomposition method involves dissolving a precursor in an organic solvent with a high boiling point, followed by a high-temperature decomposition reaction. The obtained monomers undergo burst nucleation and slow growth, resulting in the formation of monodisperse metal-based nanomaterials in the presence of surface ligands. In general, this synthesis process represents an irreversible material decomposition process devoid of oxygen involvement, which has been extensively used in the production of many pure metal nanomaterials, metal oxide nanomaterials (especially magnetic nanomaterials), bimetallic alloy nanomaterials, and metal semiconductor compounds [51]. The most common surface ligands, including oleic acid, oleylamine, oleyl alcohol, and triphenylphosphine, can affect the size and shape of metal-based nanomaterials by restricting the space and rate of crystal growth [68–70]. Singapati et al. [71] synthesized iron oxide nanomaterials using iron oleate complexes as the precursor, with oleic acid and oleylamine as ligands, and octadecene as the solvent at a high temperature of 320°C. The nanomaterials generated when using oleyl alcohol as a ligand exhibited a smaller and more irregular morphology compared to those obtained when using oleic acid. In another study, superparamagnetic iron oxide nanoparticles (SPIONs) were synthesized using oleic acid and trioctylphosphine as ligands [72]. The use of trioctylphosphine as a ligand resulted in the formation of larger-sized SPIONs compared to those obtained using oleic acid as the ligand. Liu et al. [73] successfully prepared monodisperse sea urchin-like Co-P nanomaterials by adding cobalt acetate and triphenylphosphine to an oleylamine solvent at a temperature of 290°C.

In addition to the ligand type, variables such as temperature, heating rate, inert gas flow rate, and precursor concentration used in the thermal decomposition process substantially affect the physical characteristics of the final products. Recent research has found that nucleation rates accelerate with increasing heating rates, resulting in the production of smaller metal-based nanomaterials. Additionally, a lower precursor concentration decreases the growth rate of crystals, leading to smaller metal-based nanomaterials [72, 74]. Demessie et al. [75] observed that as the nitrogen flow rate increased during the thermal decomposition reaction, iron oxide-based nanomaterials exhibited a lack of core–shell structure, decreased crystallinity, smaller sizes, and reduced magnetic heating performance.

The physicochemical properties of metal-based nanomaterials inevitably vary among different production batches. The large-scale synthesis of uniformly sized nanomaterials is necessary to minimize these differences and optimize production costs. Feld et al. [76] prepared uniform-sized SPIONs through thermal decomposition of iron oleate precursor. Iron oleate was synthesized via a simple reaction of iron carbonate with oleic acid. Iron carbonate was more stable than most iron salts, which contributed to the high uniformity of the synthesized iron oleates and SPIONs. The size and shape of SPIONs could be precisely controlled by adjusting the reaction time and the oleic acid-to-Fe ratio. Owing to the reproducibility and high flexibility of the synthesis pathway, up to 20 g of SPIONs could be produced per batch. Dong et al. [74] presented a general approach for synthesizing uniform magnetite nanomaterials by thermally decomposing iron acetylacetonate. By simply adjusting the aging temperature, precursor concentration, and amounts of surface ligands and reducing agents, the particle size can be precisely controlled. In short, due to the good biocompatibility and relatively low cost of precursors such as metal oleate complexes and metal acetylpyruvate, coupled with the high efficiency of the synthesis method and the precise control over size and morphology, metal-based nanomaterials can be produced on a large scale via thermal decomposition for clinical and biological applications.

#### Sol-gel method

The sol-gel method is widely regarded as a versatile approach for the synthesis of various nanostructures, including metal compounds (metal oxides, fluorides, sulfides, nitrides, borides, chlorides), MOFs, and metal ion-chelate complexes [77–81]. In general, the sol-gel process involves dissolving inorganic metallic salts, such as nitrates or oxalates, in water or mixing metal alkoxides with an appropriate amount of alcohol to induce the hydrolysis process and subsequent polycondensation reaction, leading to the formation of a sol-gel system with a porous structure. The subsequent interconnections among the particles in the system contribute to gel formation. Finally, the metal-based nanomaterials can be obtained with excellent purity and uniform distribution via drying and heat treatment [82]. In contrast to conventional methods, the sol-gel synthesis process does not require high-temperature treatment, offering a distinct advantage in the efficient and economic production of high-quality materials.

Properly setting working parameters, such as the precursor concentration, pH, aging time, drying speed, and calcination temperature, for the reaction is crucial because these variables affect the structure, porosity, shape, and size of the products [83–85]. In a previous study, Lal et al. [84] reported the influence of calcination temperature on the synthesis of TiO_2_ nanomaterials using the sol-gel method. The sol-gel reaction was performed using titanium tetraisopropoxide, isopropanol, and deionized water, and the obtained products were calcined at temperatures ranging from 300 to 800°C. The authors highlighted that the increase in calcination temperature led to a gradual enlargement of the crystalline size, a transformation of the crystalline phase from anatase to rutile, and a progressive reduction in the bandgap. In another study, Rodríguez-Barajas’ group evaluated the effect of variations in solution pH and precursor concentration on the size and morphology of metal-oxide nanomaterials prepared by the sol-gel method [85]. In this study, TiO_2_ nanomaterials, ZnO nanomaterials, and various mixed metal oxide nanomaterials (with TiO_2_-ZnO molar ratios of 3:1, 1:1, and 1:3) were synthesized using different concentrations of titanium (IV) butoxide and zinc nitrate as precursors, ethanol and distilled water as solvents, and adjusting the solution pH using HNO_3_ and NH_3_·H_2_O. As a result, all products synthesized under basic pH conditions were larger than those generated in the acidic process. Moreover, TiO_2_-ZnO binary oxides prepared with varying precursor concentrations displayed diverse morphologies, as evidenced by semi-globular shapes in TiO_2_-ZnO molar ratios of 3:1 and 1:1 compared to tubular rounded shapes in a molar ratio of 1:3. Also, Sheikhi et al. [86] synthesized ZnO nanomaterials using the hydrolysis and condensation reactions of a zinc acetate precursor and investigated the effects of solution pH on the final products by adjusting the pH with NaOH. As the pH of the solution increased, morphological changes were observed in the ZnO nanomaterials, with a decrease in their average diameter and an increase in their aspect ratio. The rod-like product was obtained during the aging process from 6 to 12 h, and the average diameter and length of the product increased with increasing aging time. However, the product transformed into nanoplates at an aging time of 18 h, resulting in a decrease in both average diameter and length.

Table 3 summarizes the scalability, reproducibility, and cost-effectiveness of the above synthesis strategies. In addition to the common synthesis methods for metal-based nanomaterials mentioned above, there are other noteworthy synthesis approaches, such as the prechelation-polymerization method, which represents a prevalent strategy employed in the synthesis of polydopamine-chelated metal ions. This method involves the chelation of metal ions and the oxidative polymerization of dopamine monomers in an aerobic, weakly alkaline environment at room temperature and atmospheric pressure [87]. In summary, the synthesis process is intricate and rigorous. Metal-based nanomaterials with different sizes, morphologies, excellent crystallinity, and high purity can be prepared by carefully selecting a synthesis scheme and precisely adjusting the reaction parameters. These synthetic approaches confer unique physicochemical properties to metal-based nanomaterials, enabling their use in cancer theranostics.

**Table 3:** Summary of the scalability, reproducibility, and cost-effectiveness of synthesis strategies for metal-based nanomaterials

Synthesis strategies	Scalability	Reproducibility	Cost-effectiveness
Precipitation method	Good scalability; Suitable for large-scale production	Good reproducibility; Through precise control over reaction conditions	Relatively low cost
Hydrothermal method	Fair scalability; A certain degree of scalability can be achieved by adjusting the reaction system	Good reproducibility; Through strict control over temperature and pressure	Relatively high cost; Require autoclave and specific reaction conditions
Solvothermal method	Fair scalability; Similar to hydrothermal method	Good reproducibility; Through strict control over reaction conditions, especially solvent type and temperature	Depend on the solvent used; Higher than precipitation method
Thermal decomposition method	Good scalability; Suitable for industrial-scale production	Good reproducibility; Through control over precursors and temperature	Good cost-effectiveness; Depend on the precursors and reaction conditions
Sol-gel method	Good scalability; Can be scaled up by adjusting solution composition and concentration	Good reproducibility; Through precise control over solution pH, temperature, and reaction time	Good cost-effectiveness; Require specific organic solvents

### Modification of metal-based nanomaterials

Proper surface modifications are essential for enhancing the stability, biocompatibility, tumor-targeting capability, and circulation longevity of metal-based nanomaterials for their effective application in tumor theranostics. Unmodified metal-based nanomaterials readily adsorb opsonins onto their surfaces, which makes them easily recognizable and rapidly cleared by the reticuloendothelial system (RES) [88]. Surface modification using hydrophilic polymers is the most common approach to improve biocompatibility and overcome the short half-life of blood circulation [89]. PEG possesses hydrophilic and electrically neutral properties, making it one of the most significant surface modifiers for metal-based nanomaterials. As the molecular weight of PEG increases, the length of PEG chains increases, leading to enhanced distance and spatial repulsion between the nanomaterials, resulting in improved stability [90]. In addition, the increased PEG chain length and surface density prolong the circulation time of nanomaterials in vivo. This is ascribed to the fact that the long-chain or high-density brush conformation of the PEG coating is more capable of bringing the zeta potential of the nanomaterials closer to neutrality, which can reduce opsonin adsorption onto the nanomaterials and further inhibit macrophage uptake [91, 92]. Furthermore, the dispersity of the PEG coating influences the circulation time and biodistribution of the nanomaterials. Tian et al. [93] demonstrated that, compared to polydisperse PEG-modified Au nanomaterials, monodisperse PEG-modified Au nanomaterials exhibited reduced protein adsorption, prolonged blood circulation time, and enhanced tumor accumulation. Recently, Wan et al. [94] investigated the pharmacokinetics and biodistribution of a series of ^68^ Ga-labeled NOTA-chelated bicyclic peptides, which were modified with PEG chains of varying lengths (*n* = 2, 4, 6, 12, 24). These PEGylated nanoprobes demonstrated good hydrophilicity and stability and were primarily distributed in the salivary glands, lungs, and pancreas, with renal excretion. The tumor uptake and retention of all 5 PEGylated nanoprobes were significantly enhanced compared to those of the unmodified nanoprobes. The nanoprobes coated with longer PEG chains (*n* = 12) showed greater accumulation in the tumor than those coated with shorter PEG chains (*n* = 2, 4, 6). In addition to PEG, metal-based nanomaterials functionalized with other polymers, such as polyvinylpyrrolidone (PVP), polylactide, and polyacrylic acid (PAA), also exhibit good stability and biocompatibility, as well as prolonged circulation time [95, 96].

Targeted modification is a necessary strategy to enable the accumulation of metal-based nanomaterials in large quantities at the tumor site and to exert effective imaging or therapeutic effects. Targeting methods for metal-based nanomaterials mainly include passive targeting through the enhanced permeability and retention (EPR) effect and active targeting through endocytosis, with active targeting being more effective [97]. Nanomaterials functionalized with appropriate ligands can actively target tumor cells by specifically binding to receptors that are overexpressed on the surface of these cells, thereby achieving precise and efficient tumor diagnosis and treatment. Notably, the utilization of cell membranes for surface modification has yielded significant results, not only in terms of their high biocompatibility and degradability, but also due to their “stealth” ability to avoid removal by the RES system and their homologous targeting effect that allows accumulation in tumor cells. These advantages make cell membranes highly suitable for surface modification. In summary, appropriate modification approaches can be freely selected based on the specific purposes of metal-based nanomaterials in tumor theranostics.

### Biosafety

The preceding paragraphs have primarily focused on the synthesis of metal-based nanomaterials, but their biosafety and toxicity remain significant hurdles in their transition from synthesis to clinical translation. The biosafety of metal-based nanomaterials is inextricably associated with multiple factors, including degradation kinetics, biodistribution, tissue enrichment, and excretion behavior. Many metal-based nanomaterials have relatively stable structures, leading to slow and incomplete degradation in vivo, which makes it difficult to completely remove them through hepatic or renal metabolism. The prolonged presence of metal-based nanomaterial residues within the body can interact with biochemical systems, raising toxicity concerns [98].

Nanomaterials should remain stable during the cycle and degrade naturally once they have fulfilled their functions. The design of TME-responsive degradation types is a feasible approach to achieve this goal. For example, a nanocomposite comprising zeolitic imidazolate framework-8 (ZIF-8) loaded with MnO_2_ and luminol-Au nanoclusters remained stable in physiological environments but could degrade and exert chemodynamic therapy (CDT), PTT, and immunotherapeutic effects in a weakly acidic and glutathione (GSH)-rich TME. The degraded zinc, manganese, and gold could be readily excreted from the liver and kidneys without causing harm to the major organs [99]. Additionally, to optimize the in vivo performance of metal-based nanomaterials, it is feasible to link the materials to targeting moieties such as proteins, peptides, biotins, nucleic acid aptamers, and antibodies through rational surface modifications, thereby enhancing the uptake of these materials by tumor cells and reducing their toxicity to normal tissues. For example, Pt-based drugs, which are first-line chemotherapeutic agents for the treatment of many cancer types, have poor tumor accumulation and biodistribution, as well as the potential to cause serious toxic side effects. To solve these issues, biotin-modified iodine-conjugated Pt(IV) nanomaterials (Bio-Pt-I) were synthesized to specifically bind to cancer cells overexpressing biotin receptors, leading to a striking increase in the uptake of Pt(IV) and I_3_^−^ [100]. The systemic toxicity of Pt(IV) in the circulation was very low, whereas in the TME, Pt(IV) was reduced to Pt(II), exhibiting remarkably potent anticancer cytotoxicity. Concurrently, the introduction of iodine into cancer cells not only augmented the CT imaging signal but also reversed cisplatin resistance by effectively inhibiting Bcl-2 expression. The target-modified nanomaterials exhibited a unique biodistribution and antitumor toxicity, significantly enhancing the biosafety of Pt-containing drugs. Moreover, the design of metal-based nanomaterials into ultrasmall nanostructures can optimize their metabolic behavior in biological systems. Ultrasmall nanostructures can be rapidly excreted by the kidneys after sufficient enrichment and retention at the tumor site, thereby reducing adverse effects on normal tissues. For instance, ultrasmall folate-modified AuPd alloy nanozymes (below 6 nm) were developed to catalyze ROS generation and augment tumor-specific cytotoxicity [101]. After 7 d, these ultrasmall nanozymes were efficiently cleared from the body through urine, which prevented their prolonged enrichment in vivo and enhanced their overall safety for biological applications.

In short, the use of metal-based nanomaterials in tumor theranostics inevitably leads to the introduction of toxic metals into the body, which has long been a key obstacle to their clinical translation. Although the short-term biosafety of metal-based nanomaterials can be enhanced to a certain degree through rational structural modulation and surface modification, research into their long-term chronic toxicity to organisms remains in an exploratory stage. Future investigations into the degradation mechanisms, metabolic behavior, and long-term in vivo effects of metal-based nanomaterials will help to facilitate their translation to clinical settings.

## Metal-based nanomaterials for cancer imaging

Various imaging techniques mediated by metal-based nanomaterials have shown remarkable potential for non-invasive cancer diagnosis, such as CT imaging, nuclear imaging, MRI, FL imaging, and PAI. By injecting metal-based nanomaterials that enhance the contrast between tumors and normal tissues, these imaging modalities can provide detailed anatomical information about lesions, enabling physicians to accurately delineate surgical margins [102]. In addition, it is possible to quantify the accumulation of metal-based nanomaterials at the tumor site and enhance the detection rate of small tumors and peripheral metastases by tracking the biodistribution of these nanomaterials [103]. More importantly, metal-based nanomaterials can specifically target biomarkers that are highly expressed in tumor tissues through surface modification, which is expected to provide real-time assessments of cancer occurrence and progression at the molecular level. Such microscopic information can be amplified and visualized using a variety of imaging techniques, thus providing a reliable basis for early cancer diagnosis [104]. Each imaging modality has unique advantages, as well as some shortcomings. For instance, CT imaging is characterized by high spatial resolution and multiplanar imaging, but its primary limitation lies in its poor ability to display details of soft tissue lesions. MRI exhibits superior soft tissue resolution but has relatively low sensitivity. FL imaging demonstrates high sensitivity and temporal resolution, but suffers from limited penetration depth. Therefore, it is important to select an appropriate imaging approach based on actual requirements. Multimodal imaging can integrate the benefits of multiple imaging modalities, providing valuable information for cancer diagnosis. In this section, we primarily discuss the diverse applications of metal-based nanomaterials for cancer imaging, ranging from clinical imaging modalities such as CT imaging, nuclear imaging, and MRI to basic imaging modalities including FL imaging, PAI, and multimodal imaging (Fig. 1).

### CT imaging

CT is a technology that utilizes the attenuation of X-rays by body tissues to perform radiological imaging [105]. When incident X-rays penetrate a human body, they are absorbed to varying degrees by tissues with distinct mass attenuation coefficients, leading to differences in the recorded X-ray intensity by the detector [106]. Subsequently, through signal conversion and computer image processing, a grayscale image with varying contrast levels (from light to dark) can be obtained. However, the attenuation coefficients of numerous tumor tissues are similar to those of surrounding healthy tissues, leading to a comparable grayscale appearance and posing a significant challenge for cancer diagnosis and differential diagnosis. The introduction of exogenous CT contrast agents is a highly promising approach to address this issue. These agents contain elements with higher atomic numbers and larger K-edge energies than soft tissues, resulting in significant additional X-ray attenuation, which contributes to improved soft tissue resolution [107]. In current clinical practice, iodinated small molecules, such as commercially available iohexol, meglumine diatrizoate, and iopromide, are the most widely used intravascular contrast agents for CT imaging. Unfortunately, iodine-based contrast agents typically come with several inherent limitations. First, the relatively low K-edge energy of iodine makes it inadequate for contrast enhancement [107]. Second, due to the lack of tumor specificity and short blood circulation time, high dosages are often required to improve soft tissue contrast, which increases the risk of radiation injury to patients [108]. Third, the direct toxic effects on renal tubules and the potential to induce severe allergic reactions limit their application scope to the population [109–111].

Nanomaterials composed of metals such as gold, bismuth, lanthanides, and transition metals are expected to alleviate these issues. These metal elements have larger X-ray attenuation coefficients than iodine, allowing for better CT image contrast at high tube voltages (> 100 kVp) [107]. Moreover, the surface modification of metal-based nanocontrast agents with suitable components, such as polymers, functional groups, and ligands, can provide additional advantages, including prolonged retention time in vivo, superior structural stability in the bloodstream, improved biocompatibility, and tailorable tumor targeting [112–114].

Based on their excellent X-ray attenuation coefficient and chemical inertness, Au nanomaterials possess notable advantages for contrast-enhanced CT imaging and cancer diagnosis. At the equivalent concentration, the X-ray attenuation coefficient of the Au nanomaterials is significantly greater than that of Omnipaque, a widely used iodine-based contrast agent [115]. Until now, Au nanomaterials with various morphologies, including rod-shaped [116], bone-shaped [117], spherical [118], shell-like [119], and star-shaped [120], have been reported for their application in CT imaging for cancer diagnosis. These Au nanomaterials have an excellent signal-enhancing effect in tumor tissues. Notably, the targeting ligands can be conveniently attached to Au nanomaterials through the interactions between the sulfhydryl functional groups and surface atoms of the Au nanomaterials. This simple targeted modification can significantly increase the specific concentration of Au nanomaterials at the tumor site, thereby improving CT contrast imaging [121, 122]. For instance, Wu et al. [123] directly conjugated the cyclized asparagine-glycine-arginine peptide (SH-cNGR) to Au nanomaterials via Au–S bonds. SH-cNGR-modified Au nanomaterials were able to specifically bind to CD13, which was overexpressed in the tumor angiogenesis endothelium, thus leading to efficient intracellular accumulation and high CT values in targeted tumor regions. In contrast to untargeted Au nanomaterials, SH-cNGR-functionalized Au nanomaterials showed higher cellular internalization rates and better tumor contrast enhancement, along with improved distribution within the tumor. SH-cNGR-functionalized Au nanomaterials exhibited promising potential for tumor-specific CT imaging. PEG with a sulfhydryl group at one end was employed as a linker to attach cetuximab, an antibody targeting the epidermal growth factor receptor, to the surface of Au nanomaterials [124]. Mice injected with cetuximab-targeted Au nanomaterials exhibited increased CT values at the tumor site compared to those that received injections of nontargeted Au nanomaterials. This difference was ascribed to the superior efficacy of active targeting compared to passive accumulation through the EPR effect, leading to a higher concentration of Au elements within the tumor tissue. Indeed, prolonging the circulating half-life of metal-based nanomaterials represents a viable strategy for improving the CT contrast in tumors, which can be achieved through strategic adjustments in nanomaterial size or surface modification with PEG chains. Dong et al. [121] investigated the in vivo biodistribution of PEGylated Au nanomaterials with core sizes ranging from 4 to 152 nm. They reported that, compared to larger Au nanomaterials, smaller Au nanomaterials exhibited prolonged circulation time and enhanced vascular contrast.

Bismuth-based nanomaterials exhibit higher X-ray attenuation than Au nanomaterials and have the advantages of low cost and low toxicity, making them promising candidates as CT contrast agents for cancer diagnosis. Amato et al. [125] investigated the contrast enhancement effect of pure elemental bismuth. They found that at different tube voltages, the CT image enhancement effect of bismuth was 1.35 – 2.90 times higher than that of iodine at the same concentration. In another study, Zelepukin et al. [126] synthesized BiOCl nanosheets containing up to 80% bismuth by mass. In comparison to barium sulfate, a typical contrast agent utilized in the gastrointestinal system, the generated nanosheets provided 2.55 times better CT imaging contrast with an equal dose of barium sulfate. In addition to the distinguished contrast enhancement capability of the bismuth, its sufficiently long blood circulation time must also be considered to magnify the differences between normal tissues and cancerous lesions. To address this challenge, Xu et al. [127] synthesized PEGylated Bi nanomaterials using the one-pot method. Although the incubation time was prolonged to 4 h, the internalization of the PEG-Bi nanomaterials in macrophages was significantly reduced compared to that of the Bi_2_O_3_ nanomaterials. This in vitro study indicated that the PEG coating played a critical role in helping Bi-based nanomaterials evade rapid clearance by the RES system. The team confirmed the findings through additional in vivo assessments. The PEG-Bi nanomaterials exhibited much less liver accumulation compared to the Bi_2_O_3_ nanomaterials after 1.5 h of intravenous injection, demonstrating an extended systemic circulation time. The PEG-Bi nanomaterials exhibited great capability for enhancing the contrast in CT imaging. Furthermore, although the Bi element possesses relatively low toxicity, it is necessary to modify the surface of Bi-based nanomaterials with biocompatible polymers to reduce their potential toxicity to healthy tissues. Shakeri et al. [128] developed BiOI nanomaterials with surface modifications using PVP and hyaluronic acid (HA) for cancer CT imaging. Both types of BiOI nanomaterials were separately incubated with cancerous cells. The authors found that, even at a high concentration of 4 mg/ml, both types of BiOI nanomaterials showed no notable cytotoxic effects, which was attributed to the enhanced biosafety conferred by the addition of PVP and HA. The efficient uptake of Bi-based contrast agents by malignant cells can be achieved through ligand-mediated active targeting, thus maximizing the CT contrast enhancement effect. Bao et al. [129] constructed Bi-based mesoporous-silica-coated nanomaterials (BMSNs) modified with breast cancer-targeting peptides (termed AR) for cancer imaging and further highlighted the superiority of active targeting through in vivo imaging investigations. In the study, BMSN-AR, untargeted peptide-modified BMSNs, or PEG-modified BMSNs were injected into mice with breast cancer tumors. As a result, only BMSN-AR significantly aggregated at the tumor site 1 h after injection and was retained until 24 h, with a noticeable CT contrast signal. As shown, the simple EPR effect-related passive tumor targeting ability is limited, especially for Bi-based nanomaterials with a core–shell structure, making it difficult to achieve effective accumulation in cancerous cells. In such circumstances, targeted modification is the key to solving the problem.

In addition to Au-based and Bi-based nanomaterials, lanthanide elements and transition metals with high atomic numbers and K-edge energies have been developed as CT contrast agents for cancer imaging. Ghazanfari et al. [130] prepared PAA-modified metal oxide (Yb_2_O_3_, NaTaO_3_, Dy_2_O_3_, and Gd_2_O_3_) nanomaterials and found that the X-ray attenuation efficiencies of all of the synthesized nanomaterials were considerably higher than those of the commercially available iodine contrast agent Ultravist. In vivo studies confirmed a noticeable CT contrast effect following injection of an extremely low dose of heavy metal oxide nanomaterials. Additionally, the hydrophilic polymer PAA coating enhanced the stability and biocompatibility of the nanomaterials, making these lanthanide and transition metal oxide nanomaterials highly promising as in vivo CT contrast agents. Recently, Tian et al. [131] synthesized MoWO nanobundles (NBs) using a simple solvothermal method for cancer CT imaging and investigated their in vivo biodistribution. The slope of the CT attenuation values of MoWO NBs was significantly higher than that of iopromide. Fantastic visual enhancements were observed at tumor sites 30 min post-injection, with the maximum accumulation of MoWO NBs detected in tumor regions 6 h post-injection, highlighting the potential of MoWO NBs as CT contrast agents for cancer imaging. Unfortunately, these NBs also gathered in other parts of the mice, including the head and neck. The team further utilized targeted HA for their surface functionalization and discovered that HA-labeled MoWO NBs selectively aggregated in the tumor area 24 h post-injection, which not only improved the cancer imaging effect but also circumvented potential hazards to normal tissues.

Notably, with the development of CT technology, the newly invented spectral CT has attracted wide interest. By acquiring different X-ray energy spectrum datasets, spectral CT can provide additional energy-dependent X-ray attenuation data for materials, enabling multi-parameter tumor imaging analysis [132]. Spectral CT includes dual-energy CT and photon-counting CT. Dual-energy CT uses high and low tube voltages during the scanning process to acquire two energy X-ray datasets. Depending on the photoelectric and Compton effects, it is possible to calculate the equivalent proportion of X-ray attenuation of each material relative to a base-material pair (e.g., iodine and water) to achieve material separation [133]. Unlike dual-energy CT, photon-counting CT uses a photon-counting detector to allocate incident photons into multiple energy bins based on their energy. The boundary thresholds of the energy bins can be set to closely approximate the K-edge energies of the elements [133]. Therefore, photon-counting CT can identify and quantify a wide variety of contrast agents with elements having K-edge energies within the CT energy range. Au-, Bi-, Ta-, and rare earth element-based nanomaterials are highly suitable for spectral CT imaging of tumors [132]. For example, Lei et al. [134] synthesized PEG-Ta_2_O_5_@CuS nanomaterials as spectral CT contrast agents for the detection of sub-5 mm orthotopic hepatocellular carcinoma. Compared to Ultravist, a clinical contrast agent containing iodine with a K-edge energy of only 33 keV, the K-edge energy (67 keV) of the synthesized Ta-based nanomaterials was better matched to the CT energy spectrum (40–140 keV), yielding better contrast enhancement. Furthermore, the small size of the Ta-based nanomaterials facilitated their preferential uptake in normal hepatocytes, while exhibiting low uptake in tumor regions, thereby generating a distinct inverse contrast between small hepatic tumors and healthy tissues. These Ta-based nanomaterials exhibited significant potential for spectral CT visualization of early-stage hepatocellular carcinoma. To differentiate between iodine-enhanced normal bones and osteosarcomas, which exhibited comparable attenuation in spectral CT, Li et al. [135] developed Bi_2_S_3_ nanorods with superior imaging properties compared to conventional iodine contrast agents. These nanorods showed great promise for the diagnosis of bone tumors using spectral CT.

In summary, metal-based nanomaterials with high atomic numbers and large K-edge energies can effectively enhance the localized contrast between cancerous and normal tissues, which aids in markedly improving the diagnostic accuracy of cancer in CT and spectral CT. Nevertheless, due to the increased ionizing radiation dose following the administration of high doses of metal-based nanocontrast agents and the inherent biotoxicity of metal-based nanomaterials, it is still difficult to apply them on a large scale in clinical scenarios. Further development of biodegradable TME-responsive multifunctional metal-based nanomaterials may effectively alleviate these problems, which is expected to achieve higher tumor contrast with smaller dosages and fewer side effects.

### Nuclear imaging

Nuclear imaging comprises two principal non-invasive imaging methods, namely single-photon emission computed tomography (SPECT) and positron emission tomography (PET), both of which rely on radionuclide detection. Specifically, SPECT detects gamma rays emitted during the decay of radionuclides, while PET captures two 511 keV gamma photons in opposite directions, resulting from the annihilation between positrons emitted by radionuclides and electrons within the body. Notably, although radiolabeled small molecules such as fluorodeoxyglucose reflect the level of glucose metabolism in vivo and have been widely used as tumor PET tracers, false-positive results may occur due to the lack of tumor targeting, such as in metabolically active inflammatory lesions [136]. Compared to commercial small molecules, radiolabeled metal-based nanomaterials have the advantages of surface modifiability and large labeling capacity, thus exhibiting promising prospects in the field of tumor nuclear imaging [137]. Furthermore, as the sensitivity of SPECT and PET is significantly higher than that of CT and MRI, nuclear imaging can be conducted using only low doses of radiolabeled nanomaterials, contributing to the mitigation of toxic effects. Swidan et al. [138] developed a ^99m^Tc-doped PVP-capped iron oxide (^99m^Tc-doped IO-PVP) nanotracer for tumor imaging. The radiolabeling yield of this nanotracer was as high as 95%. Benefiting from the EPR effect, the nanotracer exhibited high tumor uptake and a high target/non-target ratio, achieving effective tumor SPECT diagnosis. Moreover, Heo et al. [139] synthesized an ultrasmall ^64^Cu-labeled copper nanocluster, which was functionalized with FC131 for specific PET imaging of triple-negative breast cancer. The modification of FC131 enabled the nanocluster to exhibit enhanced tumor-targeting capability. The ^64^Cu-labeled nanocluster showed considerable accumulation in triple-negative breast cancer cells that overexpressed CXCR4, resulting in sensitive and accurate PET diagnosis.

In summary, radiolabeled metal-based nanomaterials offer the advantages of high efficiency in radionuclide labeling and targeted modification, making them suitable for effective tumor nuclear imaging. However, there are still some challenges that need to be overcome before their clinical translation, including radiolabeling stability, metal toxicity, potential radiation damage, and reproducible synthesis. The focus of future research should be on exploring robust labeling methodologies to prevent radionuclide shedding, employing radionuclides with relatively shorter half-lives for labeling purposes, and synthesizing biodegradable metal-based nanomaterials to effectively address the aforementioned challenges. This will facilitate a gradual transition from basic research to clinical translation.

### MRI

MRI is distinguished by its exceptional soft-tissue resolution, multi-directional imaging, and absence of ionizing radiation. These features are believed to enable MRI to provide a wealth of information for non-invasive cancer detection that is not accessible through other imaging modalities [140]. This is closely associated with its fundamental imaging principles. A considerable number of hydrogen protons in vivo experience energy level transitions and become magnetized when exposed to radiofrequency magnetic fields. MRI aims to capture tissue signal intensity by measuring the relaxation time of protons as they revert from the magnetized state to the initial state after removal of the radiofrequency pulses. In this process, the time required for recovery of the longitudinal and transverse magnetization intensities is referred to as the T1 and T2 relaxation times, respectively [141]. The variances in T1 and T2 resulting from diverse tissue compositions play a crucial role in achieving favorable soft tissue contrast and serve as the foundation for differentiating malignant lesions from normal tissue in MRI. Nevertheless, the relaxation time of some early-stage cancers closely resembles that of adjacent healthy tissues, which limits the diagnostic efficacy of MRI. This challenge can be addressed by introducing MRI contrast agents because they can enhance the signal differences between tissues by altering the T1 and T2 relaxation times of hydrogen protons in the surrounding tissues [142]. Specifically, owing to the markedly increased metabolic activity of cancerous lesions, their affinity for contrast agents tends to surpass that of normal tissues, leading to a notable enhancement in MRI signal intensity in the lesion area. Generally, MRI contrast agents can be simply classified as T1 or T2 MRI contrast agents based on their relaxation rate values (r_1_ and r_2_) and r_2_/r_1_ ratio. Materials with high r_1_ values and small r_2_/r_1_ ratios are suitable for application as T1 MRI contrast agents, while materials with high r_2_ values and large r_2_/r_1_ ratios exhibit enhanced contrast in T2 MR images. Materials with high values for both r_1_ and r_2_, as well as moderate r_2_/r_1_ ratios, have great potential to provide a synergistic T1/T2 contrast-enhanced effect [141–143].

Gd chelates are the most frequently used T1 MRI contrast agents in clinical practice. The 4f orbital of Gd^3+^ contains 7 unpaired electrons, leading to a large spin magnetic moment and a high r_1_ value. However, the contrast efficiency of this type of molecular contrast agent is relatively poor because of the low utilization of Gd^3+^ and the lack of tumor targetability [144]. Consequently, higher doses are required to achieve satisfactory contrast in cancerous lesions, which inevitably increases the risk of serious side effects for patients, such as nephrogenic systemic fibrosis [145]. Compared to Gd chelates, Gd-based nanomaterials possess more advantages. Nanostructures increase the payload of Gd^3+^ compared to small molecules, resulting in enhanced positive contrast in T1 MRI [146]. Surface modification of Gd-based nanomaterials can endow them with a variety of functional characteristics, including enhancing their biocompatibility and facilitating their targeted accumulation at tumor sites. As an example, Dai et al. [147] prepared PEG-coated gadolinium oxide (PEG-Gd_2_O_3_) nanomaterials for contrast-enhanced MRI of renal carcinoma. The presence of the PEG polymer not only prolonged the circulation half-life of the nanomaterials, which was 2.8 times longer than that of the commercial Gd chelator Magnevist (Gd-DTPA), but also contributed to its improved biosafety. When equal amounts of PEG-Gd_2_O_3_ nanomaterials and Gd-DTPA were injected in vivo, the signal intensity in the tumor region of the former was notably stronger than that of the latter, which was ascribed to the higher r_1_ value of PEG-Gd_2_O_3_ nanomaterials (29.0 mM^−1^ s^−1^) compared to Gd-DTPA (4.2 mM^−1^ s^−1^). PEG-Gd_2_O_3_ nanomaterials exhibited significant potential for MRI of renal carcinoma. In a recent study, Liu et al. [9] innovatively designed single-atom Gd-based nanomaterials (named Gd-SA) as a T1 MRI contrast agent for cancer imaging. Specifically, the Gd atoms were anchored on a hollow carbon nanosphere through 6 N atoms and 2 O atoms and subsequently modified with PEG. The synthesized Gd-SA nanomaterials displayed superior stability and minimal cytotoxicity. Compared to Gd-DTPA, the Gd-SA nanomaterials exhibited higher utilization of Gd atoms and larger contact areas with surrounding water molecules. As a result, the r_1_ value of nanomaterials was as high as 11.05 mM^−1^ s^−1^, enabling more precise visualization of tumor margins and better signal enhancement of tiny cancerous lesions (1 mm in diameter). Such safe and efficient single-atom Gd-based nanomaterials have tremendous potential as MRI contrast agents for early and precise cancer diagnosis.

Mn-based nanomaterials with paramagnetic properties are also promising candidates as T1 MRI contrast agents. To enhance MRI sensitivity for cancer detection, Li et al. [148] developed phospholipid-coated pH-sensitive L-epigallocatechin gallate complexed Mn^2+^ (L-EGCG-Mn) nanomaterials. They reported that as the pH decreased from 7.4 to 5.5, the r_1_ value of the prepared nanomaterials gradually increased from 1.77 to 7.23 mM^−1^ s^−1^ in human serum albumin at 3 T. After injection, conspicuous bright T1 MRI signals were observed at the tumor site, which were attributed to the disintegration of L-epigallocatechin gallate and the subsequent release of Mn^2+^ in the acidic tumor environment. These pH-responsive Mn-based nanomaterials enabled the selective enhancement of tumor tissues in T1 MRI. Nevertheless, the contrast enhancement of early-stage brain tumors remains challenging because of the blockade of the intact blood–brain barrier. To address this problem, Qin et al. [102] synthesized carbonized complexes of Mn^2+^ (named Mn@CCs) by using sealed carbonized shells to encapsulate Mn^2+^, which exhibited a remarkably high r_1_ value of 22.1 mM^−1^ s^−1^ at 9.7 T. A striking increase in brain parenchymal signal was observed after intravenous injection of Mn@CCs into healthy mice, confirming their excellent ability to penetrate the blood–brain barrier. When injected into glioma-bearing mice, ultrasmall tumors (1 mm) showed significant positive contrast enhancement with clear tumor margins, demonstrating the advantages of Mn@CCs as a T1 MRI contrast agent in the early and accurate diagnosis of ultrasmall brain parenchymal tumors. Moreover, to decrease the potential harm to biological tissues arising from high-dose T1 MRI contrast agents, Yang et al. [149] developed a series of zwitterionic dopamine sulfonate-coated MnO nanomaterials and investigated the impact of their physical properties on the sensitivity of tumor T1 MRI. As a result, the high Mn^2+^ occupancy rate on the crystal surface favored the r_1_ value, whereas the large geometric volume had an adverse influence on the T1-enhanced effect. Among the different shapes of MnO nanomaterials, MnO octahedrons exhibited the highest r_1_ value of 20.07 mM^−1^ s^−1^. After intravenous injection of the same doses of octahedral and cross-shaped MnO nanomaterials, the former demonstrated higher enrichment in the hepatic cancer tissues of mice. MnO octahedrons with an ultralow dose achieved the same T1 contrast-enhanced effect as high doses of cross-shaped MnO nanomaterials and clinically used Mn-DPDP on hepatic and subcutaneous tumors, demonstrating the significant potential of octahedral MnO nanomaterials for cancer T1 MRI and precise cancer diagnosis.

Superparamagnetic Fe_3_O_4_ nanomaterials with high magnetic moments are the most extensively studied T2 MRI contrast agents. Surface stealthiness, achieved through PEG coating, is widely employed to ensure the biocompatibility of Fe_3_O_4_ nanomaterials and provide a sufficient image-acquisition time window. The common strategy for preparing PEGylated Fe_3_O_4_ nanomaterials involves the use of anchoring groups, which have been reported to potentially provide additional T2 signal enhancement by increasing the inhomogeneity of the local magnetic field. However, this approach requires relatively complex preparation protocols [150]. Considering this situation, Thapa et al. [151] successfully prepared anchor-free PEGylated Fe_3_O_4_ nanomaterials via dipole-cation covalent interactions. These Fe_3_O_4_ nanomaterials without anchoring groups exhibited a remarkable negative contrast effect in T2 MRI, with no cytotoxic response detected in cancerous cells following incubation. Additionally, to optimize the contrast enhancement of cancerous lesions, Soleymani et al. [152] fabricated almost nontoxic FA@Fe_3_O_4_ nanomaterials by conjugating dextran-coated Fe_3_O_4_ nanomaterials with folic acid using a one-pot hydrothermal method. After repeated intraperitoneal injections of the acquired FA@Fe_3_O_4_ nanomaterials at the same dose into breast tumor-bearing mice, a gradual decrease in T2 MRI signal intensity was observed in the tumor region. This was due to the high affinity of FA@Fe_3_O_4_ nanomaterials for the overexpressed folate receptors in breast cancer, which resulted in their significant accumulation at the tumor site. In a recent study, D-glucosamine, which is highly sensitive to cancerous cells, was attached to PEGylated Fe_3_O_4_ nanomaterials, and the resulting Fe_3_O_4_ nanomaterials markedly reduced T2 MRI signal intensity [153]. These Fe_3_O_4_ nanomaterials were primarily distributed in the gastrointestinal tract 40 min after injection into normal mice. PEGylated D-glucosamine-functionalized Fe_3_O_4_ nanomaterials serve as novel T2 MRI contrast agents, providing new opportunities for the efficient detection of metabolically active tissues.

In conclusion, the use of nanomaterials based on Gd, Mn, and Fe as MRI contrast agents exhibits significant potential in the realm of cancer imaging due to their compelling properties, such as high relaxivity, facile synthesis, and tunable surface chemistry. However, there are few reports on the large-scale production of these MRI contrast agents. The relaxivity, tumor-specific distribution, stability, biosafety, and cost-effectiveness of metal-based magnetic nanocontrast agents should be considered in future studies to facilitate their clinical translation. Notably, in addition to their application in in vivo MRI, magnetic nanomaterials based on metals or metal oxides can also be used for biomarker measurement via nuclear magnetic resonance effects. The integration of diagnostic magnetic resonance platforms with magnetic nanomaterials facilitates the real-time, quantitative, and highly sensitive detection of tumor markers, which holds great promise in tumor diagnosis [154].

### FL imaging

FL imaging provides the most intuitive information for cancer lesions and is characterized by high temporal resolution and high sensitivity. Therefore, its potential application in non-invasive cancer diagnosis has attracted increasing attention. However, light with shorter wavelengths tends to interact more strongly with biological tissues and be easily absorbed or scattered in vivo, significantly limiting the penetration depth and resulting in high levels of tissue autofluorescence. The presence of background noise and poor penetration of FL imaging has severely restricted its use in cancer diagnosis and treatment. Faced with this dilemma, FL imaging in the NIR region, especially in the second near-infrared (NIR-II, 1000–1700 nm) region, is extremely attractive. NIR-II light exhibits minimal scattering and absorption by biological tissues, allowing for deeper tissue penetration depth and substantially improved spatial resolution, which opens up a broad space for the application of FL imaging in deep-located tumor detection [8, 155, 156].

Metal-based quantum dots (QDs) serve as NIR-II fluorophores, demonstrating tremendous potential in real-time and dynamic cancer imaging because of their size-tunable fluorescence emission, excellent optical stability, high quantum yield, and strong fluorescence intensity [157]. Nonetheless, metal-based QDs suffer from critical toxicity induced by exposure to heavy metals, which greatly impedes their practical application in vivo [158]. To address this issue, Awasthi et al. [159] successfully synthesized water-dispersible Ag_2_S QDs encapsulated in PEGylated polyacylthiourea dendrimer (named PEG-PATU) using a one-pot method, in which long PEG chains played a role in reducing cytotoxicity, improving biocompatibility, and prolonging blood circulation time. Furthermore, Ag_2_S QDs were compartmentalized in the dendrimer cavities, contributing to their excellent stability and outstanding capabilities in NIR-II FL imaging, thereby enabling real-time imaging of the vascular system. It is worth mentioning that NIR-II FL signals were detected in the liver within 2 min post-intravenous injection of A549 cancer cells containing Ag_2_S QDs into the mice. Subsequently, the signal gradually appeared in other parts of the body as the cancer cells were redistributed in the blood circulation, confirming the real-time tracking ability of the PEG-PATU Ag_2_S QDs for monitoring cancer metastasis. In another study on FL imaging of circulating tumor cells, Lian’s group [160] fabricated core–shell CISe@ZnS QDs by coating CuInSe_2_ with a ZnS passivation layer, resulting in a significant increase in the photoluminescence quantum yield to up to 21.8%, which was significantly higher than that of other previously reported Pb/Cd-free QDs. The CISe@ZnS QDs were then coated with hydrophilic phospholipids and conjugated with the targeted anti-EpCAM antibody. The NIR-II FL signal could be visualized in the tumor region 5 h after injection with a high signal-to-noise ratio, and the FL intensities increased gradually over time, indicating the excellent tumor-specific recognition ability of the target-functionalized CISe@ZnS QDs. Overall, these novel CISe FL probes with core–shell structures provided robust support for cancer diagnosis and real-time visualization of metastatic lesions.

Unlike conventional FL imaging mediated by heavy metal-based QDs, rare earth-doped nanoparticles (RENPs) can emit a wide range of spectra from ultraviolet (UV) to NIR when excited by NIR light [161]. By doping matrix materials with various types and concentrations of rare-earth ions, RENPs can convert lower-energy NIR-I light into higher-energy visible light through the upconversion luminescence (UCL) process or emit NIR-II light via the downconversion luminescence mechanism [162, 163]. Interestingly, the special imaging characteristics confer several unique advantages to RENPs. For instance, using low-energy excitation light can effectively minimize the risk of photodamage and avoid background autofluorescence interference from biological tissues while simultaneously enabling deep tissue penetration. Furthermore, using emitted visible light to activate PSs for the production of ROS or induce the release of loaded drugs allows for UCL imaging-guided cancer therapy [164, 165], whereas emitted NIR-II light can provide high-contrast FL imaging for deep-located tumor tissues [166]. Unfortunately, the biological applications of RENPs are often limited by their relatively low UCL intensity. This issue can be effectively resolved through the creation of core–shell nanostructures or by exploiting the LSPR properties of metal nanomaterials [167, 168]. Lv et al. [169] developed core–shell RENPs for the precise diagnosis of lung adenocarcinoma. Their team screened out nanoprobes with the highest UCL and NIR-II luminescence intensities as well as the longest luminescence lifetimes of NaYF_4_:Yb, Er, Eu@NaYF_4_:Nd (abbreviated as NYF:Eu) nanomaterials by adjusting the concentration of dopants (Eu, Ce, and Er). The lung adenocarcinoma-specific antibody was then conjugated to NYF:Eu nanomaterials to enhance their targeting ability. Upon injection into tumor-bearing mice, bright FL signals were observed at the tumor sites after 20 min and remained stable for a long time, with only weak FL appearing in the surrounding healthy lung tissue. These targeted core–shell RENPs have great potential as nanofluorescent probes for the precise diagnosis of lung adenocarcinoma and surgical navigation. In a recent study, Liang et al. [170] designed PAA-modified RENPs with multilayer core–shell structures, where NaYF_4_:Yb^3+^/Er^3+^@NaYF_4_:Nd^3+^@NaYF_4_ was the core, and hydrogen-bonded organic frameworks (HOFs) were the shell. The prepared RENPs gradually accumulated at murine tumor sites through the EPR effect and emitted NIR-II fluorescence under 808 nm first near-infrared (NIR-I) light excitation, thus providing visual imaging information for cancer diagnosis. Notably, the prepared RENPs could also emit UCL when irradiated by an 808 nm laser to activate the shell HOFs, which contributed to both PTT and PDT effects. These RENPs demonstrated exceptional biocompatibility and negligible cytotoxicity because of the surface modification of PAA. Accordingly, these multilayer core–shell RENPs, with combined optical, photodynamic, and photothermal properties, provided new opportunities for concurrent cancer imaging and therapy.

Overall, metal-based fluorescent nanoprobes, especially NIR-II nanoprobes, provide a powerful tool for real-time localized detection of primary cancers and metastatic lesions, as well as for precise surgical navigation. However, the potential off-target effects and long-term toxicity of metal-based fluorescent nanoprobes, as well as the high cost of NIR-II FL imaging systems, pose challenges for their future popularization. The design of metal-based NIR-II fluorescent nanoprobes using biodegradable materials, further modification with targeting molecules or homologous cancer cell membranes, and the development of novel instruments present promising solutions to these problems.

### PAI

In addition to CT imaging, MRI, and FL imaging modalities, the use of metal-based nanomaterials in PAI can also offer valuable information for non-invasive in vivo cancer diagnosis. As an innovative imaging technique, PAI integrates the advantages of traditional optical and US technologies, and has aroused considerable interest [171]. The PAI process involves the conversion of laser pulse energy into thermal energy, which leads to the thermal expansion of the material, consequently generating high-frequency acoustic waves detectable by an external US transducer [172]. By employing noble metal nanomaterials with excellent NIR window absorption and high PCE as exogenous contrast agents for cancer PAI, the limitation of poor tissue penetration in optical imaging can be effectively avoided, as well as images with superior contrast and high spatial resolution can be obtained.

Noble metal-based nanomaterials, especially gold nanorods (GNRs) with satisfactory NIR extinction coefficients and PCE, have been developed for cancer PAI, benefiting from the LSPR effect [173]. In a typical study, Alchera et al. [174] innovatively designed cyclic CphgisoDGRG peptide (Iso4)-functionalized chitosan-coated GNRs (named GNRs@Chit-Iso4) as a targeted PA contrast agent for orthotopic bladder cancer, where Iso4 served as the targeting ligand for α_5_β_1_ integrin overexpressed by non-infiltrating bladder cancer cells. Using mice bearing orthotopic bladder cancer as living models, several small and flat neoplastic regions displayed distinct PA signals under NIR laser irradiation after instillation with GNRs@Chit-Iso4, which were invisible in pure bioluminescence or US imaging. This result indicated that early-stage bladder cancer could be detected with unprecedented sensitivity via PA imaging based on GNRs@Chit-Iso4. These tumor-targeted GNRs provide intriguing strategies for the early and precise detection of superficial bladder lesions smaller than 0.5 mm in size. Regrettably, GNRs@Chit-Iso4 gradually settled to the bottom of the bladder during the PAI process, significantly impeding its practical application in the diagnosis of cancers located at the top or lateral sides of the bladder. Recently, their team has proposed targeted improvements [16]. Using the cross-linking reagent lipoic acid-polyethylene glycol 5-KDa-maleimide, which was attached to GNRs with a gold-sulfur bond on one end and to Iso4 with a thioether bond on the other end, they successfully constructed a novel PEGylated targeted PA probe called GNRs@PEG-Iso4. Due to the protection of PEG, the absorption spectrum of GNRs@PEG-Iso4 remained almost unchanged whether in a cryogenic environment at − 80°C, in a high-concentration NaCl solution, or in urine, and no aggregation was observed, demonstrating their excellent stability. More importantly, the products were distributed across the entire bladder and targeted to accumulate at tumor sites, showing strong PA signals even in tiny lesions measuring less than 0.5 mm after instillation. These PEG-modified targeted GNRs are expected to be powerful tools for PA imaging of early-stage bladder cancer, benefiting from their ability to overcome sedimentation defects. In another study, it was found that the light absorption peak of the GNRs could be redshifted from the red region (670 nm) to the NIR-II region (1020 nm) by tuning the aspect ratio of the GNRs and the thickness of the MnO_2_ shells, resulting in an enhanced photoacoustic contrast effect [175].

Although Au-based nanostructures have been broadly studied as PA contrast agents because of their outstanding optical properties and chemical stability, alternative metal-based nanostructures have recently emerged for cancer PAI. Nanomaterials based on other metal cores, such as Fe, Re, Bi, Mo, Co, Cu, V, Ta, Pd, Ag, and Ti, have shown strong PAI contrast in vivo [176–178]. For example, Zhang et al. [176] designed multifunctional DNA-Ag@Pd nanoclusters for cancer theranostics. Under 1270 nm laser irradiation, the tumor could be accurately located through Ag@Pd nanocluster-mediated high-contrast PA signal, which guided precise anticancer treatment. Interestingly, the DNA structures enhanced the stability and biocompatibility of DNA-Ag@Pd nanoclusters, offering a novel perspective for safe in vivo applications of nanomaterials. Zhang et al. [179] developed HASAIC nanomaterials by loading iohexol and chlorin e6 (Ce6) into hollow Ag_2_S/Ag nanoshells, which were then coated with thermosensitive phospholipids. The PA signal and photothermal ability of Ag_2_S/Ag nanoshells were activated when exposed to NIR laser irradiation. Furthermore, the loaded Ce6 could generate ROS under US excitation, synergistically enhancing the efficacy of CT/PAI-guided cancer treatment.

Despite the remarkable contrast-enhancing capability of metal-based nanomaterials in PAI, there lies a long and challenging path ahead for future studies on metal-based PA nanoprobes. Further studies should focus on optimizing the optical properties of metal-based nanomaterials to develop PA contrast agents that are highly efficient and economical. Additionally, a comprehensive examination of the biotoxicity and longevity of these metal-based nanomaterials is necessary before their clinical application.

### Multimodal imaging

Although a single imaging modality can provide valuable insights into cancer diagnosis, the inherent limitations of each imaging modality cannot be neglected. Multimodal imaging, which integrates different imaging modalities into a single nanosystem, is expected to provide more comprehensive and accurate information for precise cancer diagnosis from multiple perspectives. The rapid development of metal-based nanomaterials has provided new opportunities for multimodal imaging in cancer detection.

FL imaging is an optical diagnostic technique that provides optimal sensitivity and excellent temporal resolution, making it appealing for non-invasive cancer diagnosis and surgical navigation. Nevertheless, its biomedical applications are greatly hindered by limited penetration depth and low spatial resolution. Under these conditions, the combination of FL imaging with other imaging modalities is an intriguing and efficient method that can partially compensate for these limitations. For instance, high-resolution anatomical CT imaging in conjunction with high-sensitivity FL imaging can provide new possibilities for precise cancer detection. Wang et al. [180] prepared gold nanoclusters (AuNCs) functionalized with polyethyleneimine and found that CT/FL dual-mode imaging of prostate cancer cells could be achieved by exploiting the optical and X-ray attenuation properties of AuNCs. In another example based on the Au element, core–shell structured AuNCs-A@PAA/CaP nanomaterials were designed for dual-modal imaging-guided cancer therapy [181]. Intravenous injection of the products led to increased CT signal intensity and bright FL signals at the tumor site 10 h later. The desirable three-dimensional spatial resolution and sensitivity provide robust support for the visual diagnosis of cancer.

Apart from CT/FL imaging modalities, the combination of MRI and FL imaging is considered highly promising because of their complementary advantages. Guo et al. [17] reported a nanocomplex comprising core–shell FeGdNP-loaded indocyanine green (ICG)/glucose oxidase (GOD) conjugated to RGD2 and mPEG for MRI/FL dual-modal imaging of integrin α_v_β_3_-overexpressing cancer. The RGD2 peptide was attached to the surface of FeGdNP for active tumor targeting, while mPEG was grafted onto the end of RGD2 to inhibit the adsorption of non-cancerous cells. The nanocomplex synthesized in this study possessed a high r_1_ value of 13.166 mM^−1^ s^−1^, which was markedly higher than that of commercial Gd-DTPA (4.21 mM^−1^ s^−1^), benefiting from the outstanding magnetic properties of FeGdNP. In vivo studies revealed that the developed nanocomplex was capable of achieving high-contrast imaging and precise localization of deep-tissue tumors, as evidenced by the remarkable T1_-_enhanced effect observed in the peritoneal metastasis tumor region 12 h after injection. Additionally, the NIR FL signal at the tumor site was observed as early as 2 h after intravenous injection, and even mesenteric metastatic lesions measuring less than 3 mm could be sensitively detected. This nanocomplex provides a reliable guarantee for precise diagnosis and radical resection of peritoneal metastases via MRI/FL dual-modal imaging. Similarly, various advanced MRI/FL dual-modal metal-based nanoprobes have been developed and successfully used for in vivo cancer detection. For example, CuS/Gd_2_O_3_ nanomaterials have been utilized for imaging of glioblastoma [14], targeted mesenchymal-epithelial transition factor-modified PEGylated NaGdF_4_ has been used for specific recognition of head and neck squamous cell carcinoma [182], and Au/Gd@FA nanoclusters have been employed for accurate imaging of breast cancer [183], all of which exemplify the tremendous potential of MRI/FL dual-modal metal-based nanoprobes in cancer diagnosis.

High-resolution visualization of cancers can also benefit from the combination of FL and PAI because PAI provides high spatial resolution for detecting deep tissues. Bi et al. [184] prepared a SiO_2_@Ag nanoprobe with a satellite-type structure for FL/PA dual-modal imaging of colorectal cancer. They found that the prepared nanoprobe was activated by endogenous H_2_S, which is overproduced in colorectal cancer, resulting in the formation of AgS nanodots on the SiO_2_ surface. Upon irradiation with an 808 nm laser, a significant PA signal and intense NIR-II fluorescence were observed in the tumor area 24 h after subcutaneous injection of the SiO_2_@Ag nanoprobe. This H_2_S-triggered SiO_2_@Ag nanoprobe exhibited great prospects for application in high-resolution, high-sensitivity, and high-specificity imaging of colorectal cancer.

Compared to bimodal imaging, trimodal imaging mediated by metal-based nanomaterials tends to offer more comprehensive information for cancer diagnosis. For example, Wang et al. [103] fabricated Au/Mn nanodots conjugated with the targeted agent luteinizing hormone-releasing hormone for precise detection and real-time surgical navigation of prostate cancer. After intravenous injection of the prepared products into mice with tibial prostate cancer metastases, FL signals in the tumor regions were observed after 2 h, with significantly increased T1 MRI signal intensities and CT values. This CT/MRI/FL trimodal imaging mediated by targeted Au/Mn nanodots enabled the detection of prostate cancer and its metastases with exceptional sensitivity and accuracy, while simultaneously providing precise anatomical information regarding the lesions with remarkable soft tissue resolution. Furthermore, the FL signals remained visible continuously for up to 12 h after injection, facilitating accurate intraoperative localization and delineation of cancer boundaries and providing a reliable guide for the complete surgical removal of cancerous lesions. In addition to Au, the combination of Bi and Mn^2+^ enables trimodal cancer imaging. For instance, Wang et al. [185] designed PEG-modified nanocomposites comprising pure Bi nanomaterials and manganese phthalocyanine for trimodal imaging-guided cancer therapy. The relaxation property of Mn^2+^ was harnessed for MRI, the X-ray attenuation property of pure Bi nanomaterials was exploited for CT imaging, and the strong NIR absorption property of metal phthalocyanines was utilized for FL imaging. The synthesized nanocomposites accumulated preferentially in the tumor region via the EPR effect, allowing for satisfactory concurrent CT imaging, MRI, and FL imaging and facilitating accurate cancer diagnosis. Furthermore, based on their excellent UCL properties, high X-ray attenuation coefficients, and large intrinsic magnetic moments of rare earth ions, RENPs are expected to integrate the advantages of diverse imaging techniques, making them potential candidates for multimodal cancer imaging [186]. Xue et al. [187] synthesized NaYbF_4_:Tm^3+^/Gd^3+^ nanorods using a hydrothermal method for UCL/MRI/CT trimodal imaging of tumors. In this study, the nanorods emitted NIR light at a wavelength of 800 nm under 980 nm laser excitation, enabling the highly sensitive detection of malignant tumors as small as 5 mm. The introduction of Gd^3+^ and Yb^3+^ ions facilitated the nanorods to exhibit effective binary T_1_/T_2_ MRI contrast-enhanced effects in small tumor lesions, providing precise information on tumor characteristics while simultaneously avoiding potential interference from magnetization artifacts. Meanwhile, the high K-edge energy of Yb^3+^ endowed nanorods with the capability to provide outstanding contrast in CT images. The trimodal imaging mediated by these multifunctional RENPs efficiently overcame the limitations of a single imaging technique, offering promising prospects for highly sensitive and precise detection of small malignant tumors.

By combining different metal-based materials, nanoprobes can also perform multifunctional imaging across various modalities, including CT/MRI [188], CT/PAI [189], CT/FL/PAI [190], PET/MRI [191], and MRI/FL/PAI [192], fully leveraging the unique strengths of each modality in tumor detection. Multimodal imaging mediated by metal-based nanomaterials holds significant potential for providing real-time, comprehensive, and detailed information for monitoring the occurrence and progression of cancers, and has been effectively applied in various areas, including early cancer diagnosis [16], metastasis tracking [103], and visualization of cancerous cells [193]. However, studies on the interactions among distinct imaging components are limited. A more profound understanding of intrinsic mechanisms is essential for the advancement of multimodal imaging probes with enhanced performance. Furthermore, in the application of “always-on” multimodal imaging probes, different imaging signals may interfere with each other, affecting the accuracy and clarity of tumor imaging. Designing metal-based nanomaterials that respond internally to the TME or externally to stimuli such as light, sound, and heat is expected to enhance the control of imaging signals, allowing for their activation and deactivation on demand to prevent signal interference. Moreover, due to the large volume and complexity of data produced by multimodal imaging, advanced algorithms capable of simultaneously processing multiple imaging data need to be developed to extract comprehensive and useful information and perform accurate analyses. However, the development and optimization of such algorithms present significant challenges, primarily involving issues such as data alignment across various imaging modalities, image fusion, and the extraction and interpretation of information. Therefore, the continuous search for a balance among various imaging components, the design of stimulus-responsive metal-based nanomaterials, and the development of advanced imaging analysis algorithms could facilitate the widespread clinical use of metal-based nanomaterials in tumor imaging.

## Metal-based nanomaterials for anticancer therapy

Although routine chemotherapy and radiotherapy are currently the prevailing treatments for killing cancer cells, they often fail to completely eradicate cancers and may lead to toxicity in normal cells due to their nonspecific effects, inadequate drug concentration at tumor sites, and tumor resistance. In this case, the utilization of metal-based nanomaterials for targeted drug delivery, radiotherapy, PTT, PDT, SDT, biocatalytic therapy, IIT, and immunotherapy offers promising avenues for safe and effective cancer eradication (Fig. 1).

### Drug delivery

Currently, non-specific drug distribution, poor bioavailability, and unintentional systemic side effects have posed severe challenges to cancer treatment. In this regard, nanoscale drug delivery systems (DDSs) provide a potent opportunity to increase the therapeutic efficacy of drugs while minimizing systemic toxicity [194]. The properties of metal-based nanomaterials, such as their small size, large specific surface area, high porosity, strong adsorption capacity, storage stability, and potential biodegradability, make them ideal candidates as delivery carriers for DDS. Unlike the intact normal vasculature, the tumor vasculature is heterogeneous and is characterized by hyperpermeability. Therefore, small-sized metal-based nanomaterials can easily penetrate the disorganized tumor vascular endothelium and deliver drugs with different solubilities and sizes to the tumor site. As a result of the compromised lymphatic drainage system, drugs can be subsequently retained in the tumor interstitial matrix [195]. Drug accumulation in tumor tissue can be enhanced using metal-based nanosystems through such EPR effects. In addition, metal-based nanomaterials can be readily functionalized with targeting agents, enabling them to enter tumor cells through receptor-mediated endocytosis and exert their maximum anticancer effects at specific subcellular organelles [196]. Moreover, the design of stimulus-responsive metal-based nanomaterials, whether based on intrinsic stimuli, such as pH and enzymes, or external stimuli, like light, temperature, and magnetic fields, has been proven to achieve controlled drug release, which prevents their premature release in blood circulation and contributes to enhanced drug bioavailability [197, 198]. Moreover, the absorption of plasma proteins onto nanocarriers was shown to be reduced by surface modification with long-chain polymers such as PEG, which effectively prolonged their blood circulation time and enhanced the efficiency of drug delivery [199]. In recent years, metal-based nanomaterials have been engineered as targeted nanocarriers for distinct drugs, such as chemotherapy agents [200], nucleic acids [201], vaccines (including various antigens and adjuvants) [202], immune checkpoint modulators [203], and radionuclides [204]. Furthermore, compared to many conventional carriers, metal-based nanomedicines can not only function as carriers but also possess inherent imaging and therapeutic capabilities, making them highly promising in the field of tumor theranostics.

In terms of chemotherapeutic drug delivery, metal (e.g., Ag [205], Au [206], Cu [207], Pd [208], Pt [209]) nanomaterials, metal oxide nanomaterials [13], metal sulfide nanomaterials [210, 211], and MOFs [212] have attracted extensive attention as effective tumor-targeted nanocarriers. Ag nanomaterials have been broadly studied in the field of biomedicine due to their anticancer properties. Silver reduces the viability of cancer cells and induces cell death through DNA strand damage, ROS production, and autophagy [213, 214]. The synergistic effect of Ag nanomaterials and loaded drugs enhances cytotoxicity against cancer cells, leading to more potent cell-killing activity [205]. Thapa et al. [215] reported a nanoplatform composed of PVP-modified graphene oxide embedded with Ag nanomaterials for the delivery of methotrexate. The combination of methotrexate and Ag nanomaterials increased their cell-specific uptake in cancer cells with folate receptors and enhanced subsequent ROS generation and DNA damage, resulting in superior cancer therapeutic performance. In addition to Ag nanomaterials, other solid metal nanomaterials have emerged as promising delivery vectors because their surfaces can be conveniently conjugated with diverse functionalities, such as targeted ligands, biocompatible additives, and chemotherapeutic agents. Hongsa et al. [216] successfully synthesized a nanosystem based on collagen and biotin-quat188-chitosan (Bi-QCS) co-coated Au nanomaterials for the delivery of 5-fluorouracil (5-FU). Modification of the Bi-QCS layer can markedly enhance the encapsulation efficiency of 5-FU by increasing the surface-to-volume ratio and improving the cellular internalization of the nanosystem by enhancing the electrostatic interactions between its positive charges and negatively charged cell membranes, and facilitating biotin receptor-mediated endocytosis. The anticancer activity of 5-FU was significantly improved by incorporating Au-based nanocarriers compared to free 5-FU. Zhang et al. [197] designed a PEG-modified CuS/MoS_2_ nanoplatform for transporting doxorubicin (DOX) to the tumor site. The intracellular release of DOX triggered by acidic pH and NIR laser irradiation potentiated its interaction with cancer cells, resulting in substantial induction of cell death. Beyond pure metal nanomaterials and metal compound nanomaterials, nanoscale MOFs are also considered to be a promising class of drug vehicles. Nanoscale MOFs feature porous structures, highly tunable pore sizes, large surface areas, and good biodegradability. They can easily load diverse drugs on their surfaces or entrap agents within their pores and frameworks, thus enabling high loading content. Li et al. [217] developed a high-loading DOX nanocarrier with GNR and ZIF-8 as the core and shell, respectively. The pH- and thermo-responsive degradation of ZIF-8 allowed for the release of high doses of DOX at the specific tumor site, and the synergistic effect of DOX and GNR-induced PTT led to a remarkable tumor inhibition rate of 90%.

In addition to chemotherapy, gene therapy represents a highly promising and effective approach for cancer inhibition. However, the delivery of nucleic acids to the desired targeted site of cancer cells [small interfering RNA (siRNA) and messenger RNA to the cytoplasm, while DNA to the nucleus] remains a formidable challenge [218]. In this respect, the reasonable design of metal-based nanomaterials can protect nucleic acids from unwanted endosome/lysosome-related degradation and facilitate their release into the cytosol. Lin et al. [219] prepared ZIF-8 nanomaterials containing ICG to trap siRNAs via electrostatic adsorption. The local heat generated by ICG upon light irradiation concurrently accelerated the dissociation of pH-sensitive ZIF-8 and induced endosome/lysosomal rupture, thereby facilitating the precise cytoplasmic release of siRNA and achieving gene downregulation as well as enhanced tumor suppression. Furthermore, valid transport of substantial amounts of DNA into the nucleus is crucial for enhancing transfection efficiency. Metal-based nanomaterials functionalized with cationic organic compounds and nuclear localization signal (NLS) peptides exhibit remarkable potential as effective vehicles for specific DNA delivery [220]. Zhao et al. [221] synthesized a targeted nanoplatform (FA-PEG-Pam/CaP/NDs) based on calcium phosphate nanomaterials modified with NLS peptides to load plasmid DNA (pDNA), followed by coating a layer of FA-PEG-pamidronate. After internalization by cancer cells via folate receptor-mediated endocytosis, the nanoplatform underwent quick dissolution within the endosomes, facilitating pDNA escape through endosome rupture induced by Ca^2+^-mediated increased osmotic pressure. The pDNA was then efficiently delivered into the nucleus with the help of NLS peptides, achieving remarkable efficacy of gene therapy both in vitro and in vivo.

Regarding cancer immunotherapy, various metal-based nanomaterials have been engineered as vectors for antigens, adjuvants, and immune checkpoint modulators to facilitate effective targeted delivery and enhance therapeutic efficacy. The cancer immune response encompasses the following key sequential events: 1) release of antigens from necrotic or apoptotic cancer cells; 2) presentation of tumor-derived antigens by antigen-presenting cells (APCs), such as macrophages and dendritic cells, to major histocompatibility complex; 3) activation of immature T cells by activated APCs; and 4) targeted recognition and elimination of cancer cells by effector T cells [222]. However, during tumorigenesis, several steps of cancer immunity may be impeded, ultimately leading to immunosuppression and evasion of immune surveillance. The efficient delivery of immunomodulatory agents can contribute to the restoration and upregulation of the cancer-immunity cycle. For example, tumor antigen-conjugated chitosan-coated CuO nanomaterials have been synthesized to activate macrophages, resulting in the elicitation of CD4^+^ and CD8^+^ T cell-mediated antitumor responses [223]. Notably, although chemotherapeutic drugs can directly kill cancer cells, they can also trigger the upregulation of immune-suppressing gene expression, which affects the final therapeutic effect [224]. The co-delivery of chemotherapeutic drugs and immunomodulators to the tumor site using metal-based nanomaterials represents an effective strategy to address this challenge. For example, An et al. [225] developed a multifunctional nanoplatform for tumor elimination. The nanoplatform (Cu@MIL-101@PMTPC) consisted of MOFs loaded with cisplatin, immunosuppressive enzyme inhibitors (1-methyl-_D_-tryptophan), photosensitizers, CaO, and Cu nanomaterials. Targeted delivery and controlled release of cisplatin in Cu@MIL-101@PMTPC effectively eliminated tumors while minimizing systemic toxicities. The presence of 1-methyl-_D_-tryptophan significantly mitigated cisplatin-induced immunosuppression, thereby enhancing the therapeutic effects. The nanoplatform could also trigger catalytic reactions to generate large amounts of ROS and activate immune responses in the TME, which achieves excellent results in effectively killing tumor cells and inhibiting their growth. Moreover, the utilization of nanomaterials for co-delivery of antigens and adjuvants, such as cyclic GMP-AMP synthase (cGAS)-stimulator of interferon genes (STING) agonists, monophosphoryl lipid A, cytosine-phosphate-guanine oligodeoxynucleotides (CpG-ODN), and 5′-triphosphate RNA (3pRNA), can significantly enhance vaccine efficacy and augment antitumor immune responses. Hou et al. [226] developed an Al-based nanosystem, namely NP-3pRNA-CpG, for the targeted co-delivery of antigen ovalbumin and adjuvants (CpG-ODN and 3pRNA) to APCs in the draining lymph nodes. NP-3pRNA-CpG has been shown to significantly enhance antigen cross-presentation with the help of an adjuvant combination through pathogen-associated molecular patterns recognized by distinct pattern recognition receptors, promoting both robust humoral and cellular immune responses to effectively inhibit tumor growth. Furthermore, given that immunosuppressive signals in the TME, such as programmed death-ligand 1 (PD-L1) and cytotoxic T-lymphocyte-associated protein 4, tend to impede T-cell activation, targeted delivery of immune checkpoint modulators based on nanosystems is expected to provide a highly efficient approach for enhancing anticancer immunity [227]. Based on this, Chiang et al. [228] created fucoidan-dextran-modified magnetic SPIONs to transport anti-PD-L1 and T cell activators (anti-CD3 and anti-CD28) for localized cancer immunotherapy. The synthesized nanosystem can specifically accumulate in tumor tissues with the aid of magnetic navigation, inducing enhanced antitumor effects through effective T-cell activation and PD-L1 pathway blockade, while mitigating off-target systemic side effects in vivo.

These metal-based nanomaterials, which deliver targeted therapeutic formulations to tumor tissues, provide a highly precise and efficient strategy for cancer treatment, significantly advancing the progress of inorganic nanomedicine within the biomedical field. However, because of the complex biological milieu, the pharmacokinetic attributes and cumulative effects of metal-based DDS are not yet clearly understood and warrant further attention. In terms of drug absorption, the bioavailability of intravenous dosage forms is superior to that of oral dosage forms [229]. Notably, Sindhwani et al. [97] demonstrated that the primary mechanism responsible for the uptake and enrichment of nanomaterials in tumor cells is through trans-endothelial pathways rather than relying on the EPR effect. The active process of cellular uptake can be influenced by various factors, including the size and surface charge of the metal-based DDS. It is widely accepted that the smaller the diameter of the metal-based DDS, the higher the extent and rate of cellular internalization. Positively charged metal-based nanocarriers are easier to internalize into cells than negatively charged or neutral nanocarriers [197]. Ouyang et al. [230] showed that surpassing the dose threshold of nanomaterial administration could facilitate enhanced tumor uptake and enrichment, with improved therapeutic outcomes. Therefore, future research should focus on developing technologies that utilize active transcytosis to enhance delivery efficiency and employ nanomaterials at doses exceeding the dose threshold for effective drug delivery. For drug distribution, the fundamental physicochemical properties of metal-based DDS, targeting strategy, extent of binding to plasma proteins, and characteristics of blood flow all exert significant influences. Actively targeted, positively charged rod- or spherical metal-based DDS have higher tumor distribution coefficients. By increasing local tumor blood flow, the efficiency of tumor-targeted delivery can be dramatically increased. In terms of healthy organ distribution, metal-based DDS primarily accumulate in the liver, kidneys, and spleen [231]. In addition, nanomaterials with sizes ranging from 10 to 200 nm are primarily excreted through the feces. Nanomaterials smaller than 8 nm can be cleared by the kidneys, while those of larger sizes can be removed by hepatic Kupffer cells [231]. Therefore, to enhance the tumor delivery efficiency and meet clinical needs, it is essential to thoroughly consider the various factors influencing the in vivo pharmacokinetics and biodistribution of metal-based DDS during their future design processes.

### Radiotherapy

Radiotherapy is one of the most important treatment methods for localized cancers. Ionizing radiation can trigger a cascade of intricate reactions in cancer cells. High-energy photons or charged particles can not only directly induce DNA damage through ionization but also interact with water molecules to generate ROS, which indirectly leads to the rupture of cellular DNA or other biomolecules and subsequently causes cell death [232]. However, the hypoxic TME leads to lower ROS production within the tumor region, thereby compromising the cytotoxic efficacy of radiotherapy. Therefore, high doses of ionizing radiation are typically required to effectively inhibit tumor growth, but they also simultaneously induce severe side effects on surrounding normal cells. The introduction of radiosensitizers into tumor tissues is expected to enhance the radiosensitivity of cancers, ensuring therapeutic efficacy while reducing radiation dosage [233]. Metal-based nanomaterials with high atomic numbers are appealing as radiosensitizers. Compared to biological tissues, nanomaterials based on high-atomic-number metals such as Au, Ag, Pt, Fe, Cu, W, Hf, Bi, and lanthanides exhibit enhanced X-ray absorption capacity, which enables increased radiation dose deposition at the tumor site. These metal-based nanomaterials can interact with radiation to emit photoelectrons, Auger electrons, and secondary low-energy electrons through processes such as the photoelectric effect and Compton scattering, ultimately inducing DNA damage in tumor cells [234, 235]. Moreover, metal-based nanomaterials can also increase cancer radiosensitivity through other pathways, including facilitating ROS production, transferring cancer cells into the sensitive G2/M phase of the cell cycle, and improving cancer hypoxia [236, 237].

Ma et al. [238] prepared PEG-modified Au-based nanostructures with diverse shapes (spherical, rod-like, and spike-like shapes) but comparable average diameters (approximately 50 nm). These Au nanostructures enhance the efficacy of radiotherapy by inducing ROS production and cell cycle arrest. Among them, spherical Au nanomaterials exhibited the largest cellular internalization amount and correspondingly had the highest sensitization enhancement ratio, highlighting the influence of the shape of Au-based nanomaterials on radiosensitization. Dou et al. [239] synthesized a series of Au nanomaterials with sizes ranging from 3 to 50 nm and found that Au nanomaterials with a size of 13 nm had optimal X-ray attenuation capability and excellent radiation sensitization effect. Notably, it has been reported that Ag nanomaterials possess higher radiosensitizing activities than Au nanomaterials at equal mass and molar concentrations [240]. In addition, transition metals such as Fe, Cu, Mn, and W can catalyze the overexpressed H_2_O_2_ in the TME to generate ROS via Fenton or Fenton-like reactions without requiring O_2_. Inspired by this, Afifi et al. [241] successfully designed a hybrid nanostructure (IO@Ag nanomaterials) comprising iron oxide nanomaterials and Ag nanomaterials. IO@Ag nanomaterials could not only reduce cellular autophagy but also release Fe^2+^ to catalyze the generation of ROS, leading to enhanced oxidative stress and DNA damage in cancer cells. The outstanding radiosensitizing activity of the IO@Ag nanomaterials significantly improved the therapeutic effect even under low-dose radiation, while minimizing the potential harm to healthy tissues compared to high-dose radiation treatment. Nanomaterials based on high-atomic-number Pt have also been used for sensitizing radiotherapy. Li et al. [18] showed that hollow PtCo nanospheres could potentiate cancer radiosensitivity by increasing radiation energy deposition, upregulating ROS generation, inducing G2/M arrest, intensifying DNA damage, impeding DNA damage repair, and catalyzing in situ O_2_ generation from H_2_O_2_. Furthermore, Hf-based nanomaterials have been reported to improve the efficacy of radiotherapy due to Hf’s exceptional properties, such as its high X-ray attenuation coefficient and chemical inertness. Fu et al. [242] immobilized HfO_2_ nanomaterials onto the surface of TME-responsive MoS_2_ nanosheets and then modified them with dextran to obtain biodegradable M/H–D. The acid-triggered degradation and photothermal heating properties of MoS_2_ promoted the internalization of small-sized HfO_2_ nanomaterials by cancer cells and accelerated the rate of peroxidase (POD)-like catalytic reactions. Under NIR and X-ray irradiation, the TME-responsive M/H–D destroyed redox homeostasis and induced severe double-strand DNA breaks in cancer cells, achieving remarkable radiosensitizing effects and efficient cancer inhibition while sparing healthy cells. Similarly, Gupta et al. [243] reported that HfO_2_@Au core–shell nanomaterials could sensitize radiotherapy by enhancing intracellular ROS generation and inducing DNA damage. Notably, phase III clinical trials investigating the radiosensitizing effect of hafnium oxide nanomaterials on soft tissue sarcomas have been completed. These nanomaterials, known as Hensify®, were approved by the European market in 2019 [244]. Interestingly, lanthanide elements can deposit additional radiation doses at the tumor site while simultaneously exhibiting great capabilities in MRI (Gd) and radioluminescence (e.g., Ce, Eu, and Y), presenting a novel strategy for precise cancer theranostics. For example, Luchette et al. [245] discovered that sub-5 nm Gd-based nanomaterials (AGuIX) possess the capability to amplify the radiation dose in HeLa cells. In further studies, AGuIX has been found to sensitize radiotherapy while exhibiting excellent contrast-enhanced MRI effects on tumors in various tumor-bearing mouse models [246]. Verry et al. [247] conducted a phase I human clinical trial of AGuIX in patients with multiple brain metastases. AGuIX can be retained in brain metastases for an extended period, providing a prolonged therapeutic window for MRI-guided radiosensitization, resulting in significant clinical benefits. Recently, Du et al. [248] developed a nanoplatform (MnCO-Tw-SCNPs) incorporating scintillating LiLuF_4_:Ce^3+^ and UV-responsive Mn_2_(CO)_10_ for cancer radiosensitization. Under X-ray irradiation, LiLuF_4_:Ce^3+^, which is radioluminescent, emits UV light to drive the photolysis of surrounding Mn_2_(CO)_10_ into CO and MnO_2_. The synergistic effect of CO-induced glycolytic inhibition and Mn^2+^-mediated Fenton-like catalytic reaction significantly potentiates DNA damage and mitochondrial dysfunction, leading to a reinforced therapeutic outcome of X-ray-activated cancer therapy.

Despite the multifaceted mechanisms by which metal-based nanomaterials can enhance the therapeutic efficacy of cancer radiotherapy, several challenges remain. Metal-based nanomaterials metabolize slowly in vivo, which is beneficial for radiotherapy, but may increase the risk of harm to the organism. Therefore, designing green and biodegradable metal-based nanomaterials and increasing their metabolic rate are issues worthy of attention. In addition, considering the influence of size and shape on the radiosensitization performance of metal-based nanomaterials, future studies should incorporate these factors into the design of metal-based nanomaterials to optimize their radiosensitizing activities for different cancer types.

### PTT

PTT is an innovative cancer therapeutic technique that employs photothermal agents (PTAs) to convert light energy into sufficient heat, thereby inducing cancer cell death. Currently, the major laser stimulus for PTT is within the NIR region, which can be further divided into the NIR-I region (750–1000 nm) and the NIR-II region (1000–1700 nm). Compared to the NIR-I biowindow, the NIR-II biowindow has a deeper tissue penetration depth and higher maximum permissible exposure, significantly advancing the safety and effective application of PTT in cancer treatment. Typically, metal-based nanomaterials with properties including LSPR, excellent NIR light absorption (especially in the NIR-II region), and high PCE are suitable for use as PTAs, such as noble metal nanomaterials [249–251], metal compound nanomaterials [252, 253], and MOFs [217]. Furthermore, excellent photothermal stability, biocompatibility, and minimal toxicity are significant prerequisites for utilizing metal-based PTAs in biomedical applications.

Zhao et al. [250] reported bovine serum albumin (BSA)-modified AuNRs with a large aspect ratio for anticancer PTT. AuNR@BSA with a size ≤ 100 nm could efficiently penetrate the blood vessels and accumulate in tumor tissues. Moreover, the reported AuNRs showed excellent tumor thermal ablation effects even under low-intensity NIR-II 1064 nm laser irradiation, benefiting from their eminent photothermal properties. Interestingly, the BSA coating led to a significant reduction in the cytotoxicity of AuNRs and a marked improvement in their biocompatibility, thereby broadening the potential utility of AuNR@BSA for in vivo cancer therapy. Li et al. [254] developed a novel nanocomposite (DMSN-Au-Ru) composed of Au, Ru, and mesoporous silica nanomaterials for tumor therapy. The incorporation of Au and Ru nanomaterials endows the DMSN-Au-Ru nanomaterials with significant potential for effective photothermal therapy. Moreover, DMSN-Au-Ru nanomaterials not only catalyzed the conversion of glucose into H_2_O_2_ through the GOD-like activity of Au nanomaterials but also decomposed H_2_O_2_ and generated singlet oxygen (^1^O_2_) with the help of Ru nanomaterials, thereby augmenting the therapeutic efficacy against tumors. Recently, Xiong et al. [255] fabricated Bi_19_S_27_I_3_ nanorods as highly efficient PTAs for cancer therapy. The prepared Bi-based nanorods exhibited exceptional PCE in both the NIR-I and NIR-II biowindows, thus contributing to their distinguished photothermal antitumor capability. Importantly, hyperthermia ablation induced by Bi_19_S_27_I_3_ nanorods under 1064 nm laser irradiation completely eradicated the cancerous lesion, offering an excellent prospect for effective photothermal cancer therapy. Geng et al. [256] developed a CuS-embedded Cu-MOF (CuS@Cu-MOF) nanoplatform for multifunctional cancer therapy. Under NIR laser irradiation, the CuS@Cu-MOF nanoplatform exhibited an outstanding photothermal effect because of its strong NIR light absorption and high PCE. The rate of the Cu^2+^-driven Fenton-like reaction could be dramatically enhanced by increasing the temperature. Furthermore, the abundant DOX encapsulation within the MOF structure could be selectively released in the acidic TME, synergistically enhancing the therapeutic efficacy of the CuS@Cu-MOF nanocomposites.

Although metal-based nanomaterials have shown potent PTT efficacy in suppressing tumor growth and inducing irreversible necrosis of cancerous cells at temperatures above 49°C, it is noteworthy that the leakage of intracellular contents may trigger deleterious inflammatory responses within tissues and stimulate tumor neoangiogenesis. In contrast, PTT at a temperature of approximately 43°C primarily induces apoptosis in malignant cells without causing inflammatory damage. However, its therapeutic efficacy remains limited. Therefore, it is crucial to optimize the morphology and structure of metal-based nanomaterials and precisely adjust the laser dosage to effectively modulate the temperature of PTT to enhance the inhibition of tumor growth and recurrence [257–259].

### PDT

In addition to PTT, PDT is a highly promising light-induced cancer treatment. The photodynamic reaction involves two mechanisms. After absorbing photons at the appropriate wavelengths, the PSs undergo a transition from their ground state to an excited singlet state. The excited singlet PSs can either emit fluorescence to release energy and return to their ground state or go through the process of intersystem crossing to convert into the excited triplet state. Hydrogens or electrons can then be transferred between the excited triplet PSs and substrates such as cancer tissues to form cation or anion radicals. These radicals can subsequently interact with O_2_ and finally lead to the generation of ROS (type I photodynamic reaction). Alternatively, the triplet-excited PSs can directly transfer their energy to O_2_ in their ground triplet state, facilitating the production of ^1^O_2_ (type II photodynamic reaction) [260]. These ROS generated at tumor sites through both pathways can ultimately induce tumor autophagy, apoptosis, necrosis, and destruction of tumor microvasculature, thereby contributing to effective tumor control [261]. Traditional PSs such as porphyrins, porphyrinoid compounds, phthalocyanines, and other derivatives are commonly employed for PDT [262]. However, the relatively low ROS quantum yield, potential inactivation in the blood circulation, and nonspecific distribution of these organic PSs significantly compromise the efficacy of PDT. Improving PS enrichment and ROS generation efficiency in tumor tissues is crucial for achieving satisfactory PDT effects. The emergence of metal-based nanomaterials, which can serve as PS vehicles, function as PSs, and act as energy transducers in PDT, has provided a promising approach for this.

To date, metal-based nanomaterials in various formulations have been reported to transport PSs. To be specific, metal nanomaterials, metal oxide nanomaterials, metal sulfide nanomaterials, MOFs, and rare-earth-doped upconversion nanoparticles (UCNPs) have shown potent potential in the delivery of PSs [263–267]. They possess the capacity to deliver a substantial payload of PSs to tumor cells through encapsulation or conjugation, preventing their early release and degradation, and achieving excellent antitumor outcomes in PDT. Among them, plasmonic nanostructures such as Au and Ag nanomaterials have garnered significant attention because of their intrinsic LSPR properties, which can increase the excitation rate of loaded PSs by enhancing the surrounding electric field [268, 269]. Yin et al. [264] reported GNR-based nanodumbbells with mesoporous silica coated at their two poles to load ICG molecules (Au@mSiO_2_-ICG). The enhanced electric field of GNRs upon NIR light irradiation greatly improved the efficiency of triplet energy transfer from ICG to O_2_, thereby amplifying the production yield of ^1^O_2_. Concurrently, the presence of GNR and mesoporous silica conferred protection against photobleaching and ensured the exceptional photostability of ICG. Therefore, Au@mSiO_2_-ICG nanodumbbells exhibit considerable promise for continuous ^1^O_2_ generation and enhanced photodynamic effects. Crous et al. [265] designed antibody-modified Au nanomaterials that could enhance the cellular uptake and intracellular concentration of the conjugated PS through both passive and active targeting mechanisms to improve PDT efficiency for lung cancer stem cells. Furthermore, metal oxide nanomaterials, such as iron oxide nanomaterials, have been investigated for their potential use in loading PSs owing to their reduced toxicity and magnetic targeting properties compared to pure metal nanomaterials. Li et al. [266] integrated MRI and FL imaging with targeted PDT by constructing PEG-modified iron oxide nanoclusters (IONCs) loaded with PS Ce6. Small-sized IONCs-PEG-Ce6 can be readily trapped by tumor tissues under the guidance of an external magnetic field, facilitating concurrent tumor targeting, monitoring, and treatment. Moreover, researchers have engineered nanoscale MOFs in which PSs such as porphyrin and its derivatives serve as organic ligands directly attached to the metal nodes, ensuring high loading and stability of PSs [267]. Notably, PSs excited by visible light face limitations in achieving effective deep-seated PDT due to their inadequate penetration ability. The development of rare-earth-doped UCNPs presents a novel approach for addressing this issue. They can not only function as delivery carriers of the PSs but also facilitate the conversion of NIR excitation light into visible light, thereby effectively activating the loaded PSs. For instance, Buchner et al. [270] synthesized rare-earth-doped UCNPs loaded with the PS Rose Bengal (RB) for NIR light-induced cancer PDT. In this study, RB was covalently bound to the surface of NaYF_4_:Yb,Er,Gd@NaYF_4_ core–shell UCNPs via a biocompatible short L-lysine. This strategy resulted in a short distance between the RB and the UCNP core, thereby enhancing the efficiency of ^1^O_2_ generation and PDT cell killing. Overall, metal-based nanomaterials play an important role in improving the cancer-targeted distribution, concentration, and ROS quantum yield of loaded PSs, providing a promising avenue for eliciting more efficacious PDT responses against cancers in the future.

Due to their inherent photocatalytic activity and photosensitizing capabilities, some metal-based nanomaterials can also act as PSs. Currently, extensive studies have been conducted to investigate the use of nanomaterials based on Au, Ag, TiO_2_, and ZnO as PSs for PDT. Compared to traditional PSs, Au and Ag nanomaterials are characterized by superior photostability and favorable water solubility, along with a high ROS generation ability, benefiting from their SPR property, which promotes energy transfer to O_2_. Zhang et al. [271] fabricated uniform 2D Au nanosheets using an ionic layer epitaxy method for anticancer applications. The large surface area and uniform ultrathin morphology of Au nanosheets facilitated robust ^1^O_2_ generation by Au surface plasmon resonance when exposed to NIR light, resulting in a remarkable ability to eradicate cancer cells, with an efficiency of 75%. Similarly, the BSA-Ag_13_ nanocluster composed of 13 Ag atoms developed by Yu et al. [272] has also been found to be feasible as a PS for anticancer PDT, exhibiting an outstanding ^1^O_2_ quantum efficiency of 1.26, which surpassed that of the majority of commercially available PSs. In addition, the low toxicity, excellent biocompatibility, photocatalytic activity, and semiconductor features of TiO_2_ and ZnO nanomaterials have positioned them as promising PSs. However, the excitation of electrons in the valence band of TiO_2_ and ZnO, the production of electron–hole pairs, as well as subsequent ROS generation, necessitate high-energy UV light with a short wavelength due to the wide energy gap band, imposing significant limitations on their practical application in deep-seated PDT [273, 274]. By incorporating metal dopants, semiconductor QDs, or carbon-based nanomaterials, the band gap of TiO_2_ and ZnO nanomaterials can be effectively narrowed, thereby broadening their spectral response range and enhancing their photochemical properties [273–275]. Yang et al. [276] employed Au cluster-modified black anatase TiO_2_ nanotubes under visible light irradiation to amplify ROS generation in cancer PDT. The nanoplatform demonstrated superior photodynamic activity and a broader range of light responses compared to pristine anatase TiO_2_ due to its ability to efficiently separate electron–hole pairs and prevent recombination. Notably, the currently reported PDT systems are predominantly based on type II photodynamic reactions, which heavily depend on the O_2_ concentration within tumor tissues. However, continuous O_2_ consumption by PSs and the hypoxic TME significantly limit ROS production during PDT, making it challenging to completely suppress tumor growth. Therefore, developing metal-based Type I PDT systems that are less affected by the O_2_ content or integrating PDT with other therapeutic approaches in the future may yield better anticancer efficacy.

### SDT

Similar to the procedure of PDT employing PSs, SDT is recognized for activating sonosensitizers through low-frequency and low-intensity US to initiate ROS generation to eliminate cancer cells. The deep tissue penetration capability of US enables efficient treatment for deeply buried tumors using SDT, which confers a significant advantage over PDT triggered by light [277]. However, the exact mechanisms of SDT in anticancer applications have not been clearly defined so far. It is widely accepted that the reliable mechanisms of SDT involve ultrasonic cavitation and ROS production. Specifically, the interaction between US fields and sonosensitizers induces periodic pressure oscillations in the liquid medium, leading to the formation, growth, and explosion of gas bubbles, a process known as ultrasonic cavitation [277]. Generally, this effect can be categorized into stable and inertial cavitation. The inertial form refers to the process of rapid bubble expansion and collapse, during which sonochemical effects, including sonoluminescence or pyrolysis of sonosensitizers, can be triggered to generate ROS, causing damage to the cell membrane and ultimately resulting in cancer cell apoptosis [278, 279].

Several traditional PSs, such as porphyrins [280] and porphyrin derivatives [281], as well as anticancer drugs such as DOX [282] and curcumin [283], have also been found to serve as sonosensitizers, but they suffer from the risks of aggregation and premature degradation in the blood [20]. In this regard, the introduction of metal-based nanomaterials as carriers has the potential to change the biological behavior of these organic sonosensitizers, protecting them from inactivation, increasing their accumulation in tumor tissue, and enhancing the therapeutic efficacy of SDT. Some metal hollow nanostructures [179], metal nanomaterials [284], bimetal nanomaterials [284], and metal oxide nanomaterials [285] have been investigated for the delivery of sonosensitizers through encapsulation or covalent conjugation. For example, Zhao et al. [286] developed hemoporfin-Cu_9_S_8_ nanomaterials and coated them with CT26 cell membranes (H-Cu_9_S_8_@CCM). The hollow structure of Cu_9_S_8_ nanospheres allowed the loading of the sonosensitizer hemoporfin with a high encapsulation efficiency of 82.6%. Capitalizing on the biocompatibility and homologous targeting ability of the CT26 cell membrane, H-Cu_9_S_8_@CCM nanomaterials demonstrated a strikingly prolonged blood circulation time and enhanced tumor accumulation. Moreover, H-Cu_9_S_8_@CCM nanomaterials could perform synergistic PTT/SDT, benefiting from the photothermal properties of Cu_9_S_8_ and the sonosensitive nature of the loaded hemoporfin. Combining these advantages, the H-Cu_9_S_8_@CCM nanomaterials exhibit unparalleled potential for inhibiting tumor growth. In addition to encapsulation, the conjugation of sonosensitizers with metal nanomaterials has been studied for antitumor SDT. Because of the rough surface of nanomaterials, the introduction of nanomaterials into the liquid medium can provide nucleation sites for the formation of cavitation bubbles and facilitate bubble collapse, which augments ultrasonic cavitation and improves the activation of sonosensitizers, leading to enhanced sonodynamic activity [287]. Sazgarnia et al. [288] synthesized an Au nanomaterial-protoporphyrin IX conjugate to improve the cancer response to SDT. Beyond promoting the cellular uptake of protoporphyrin IX, the presence of Au nanomaterials could also prolong the nonradiative relaxation time of protoporphyrin IX and facilitate the formation and collapse of cavities, thereby favorably activating protoporphyrin IX. Consequently, the utilization of Au nanomaterials as carriers for delivering protoporphyrin IX demonstrates significantly enhanced antitumor efficacy when exposed to US compared to the use of protoporphyrin IX alone. Furthermore, as discussed above, MOFs with a high porosity and large surface area are highly suitable carriers for efficient agent delivery, and their porous structure enables rapid ROS diffusion, which enhances the efficacy of SDT. In addition, porphyrin-based MOFs formed by metal ions coordinating with bridging ligands, such as hematoporphyrin and its derivatives, have also shown remarkable sonodynamic activity [289]. Based on this, Zhang et al. [290] prepared a MOF-based dual-sonosensitizer nanoplatform for application in SDT against hypoxic cancer. In this platform, the oxygen-independent sonosensitizer 2,2-azobis[2-(2-imidazolin-2-yl)propane] dihydrochloride (AIPH) was successfully encapsulated into a Zr-MOF with a porphyrin-based structure (Zr-MOF@AIPH). Under US irradiation, AIPH decomposed to produce alkyl free radicals, whereas Zr-MOF could generate ^1^O_2_, thereby significantly enhancing the antitumor efficacy of SDT in both hypoxic and normoxic environments. Moreover, the release of nitrogen during AIPH decomposition could enhance the permeation capacity of Zr-MOF@AIPH into tumor tissues through the ultrasonic cavitation effect. Hence, this MOF-based dual-sonosensitizer nanoplatform is expected to be a potent approach for treating hypoxic cancer. In conclusion, the use of metal-based nanomaterials as carriers for sonosensitizers can augment the therapeutic effect of SDT by reducing the cavitation threshold, facilitating sonosensitizer activation, and improving their tumor penetration and accumulation.

Apart from their role as carriers, metal-based nanomaterials, including noble metals (Au, Ag, and Pt), transition metal (Ti, Mn, Zn, Cu, Fe, Ce, and Al) compounds, MOFs, and metal complexes, have been extensively explored as sonosensitizers themselves. Among them, inspired by the remarkable photosensitizing capability, metal-based semiconductor nanomaterials such as TiO_2_, MnO_2_, and ZnO have been found to effectively respond to US and produce ROS for antitumor SDT [291]. However, the limited ROS generation yield of pure semiconductor nanomaterials resulting from the rapid recombination of electron–hole pairs has impeded their further application in cancer cell eradication. Several strategies have been investigated to improve the efficiency of these semiconductor nanosonosensitizers in SDT. The combination of noble metal materials with semiconductor nanomaterials provides an effective approach to address this issue. Noble metal materials can act as electron traps to avoid the recombination of electron–hole pairs, thereby enhancing the ROS generation efficiency. Based on this, Perota et al. [292] prepared Au/TiO_2_ nanocomposites as a new type of nanosensitizer for synergistic SDT and PTT against melanoma. Upon exposure to the US, the valence band electrons of TiO_2_ can be excited to leave the positive holes and be received by Au with a relatively lower Fermi level. This process effectively facilitates electron–hole separation and inhibits their recombination, leading to a considerable increase in ROS generation, ultimately causing enhanced melanoma cell apoptosis. Similarly, the TiO_2_ nanosheets with Au nanocrystals grown on their edges (Au-TiO_2_ nanosheets) developed by Cao et al. [293] exhibited substantially enhanced ROS generation efficiency compared to pure TiO_2_. Moreover, after modification with triphenylphosphine and the AS1411 aptamer, which specifically target mitochondria and the cancer cell membrane, respectively, the synthesized Au-TiO_2_ can efficiently accumulate in the mitochondria and achieve the superior antitumor efficiency of SDT to completely suppress tumor growth. In addition to the metal-coupling strategy, the rational design of defect-rich metal-based semiconductor nanostructures can motivate the efficient separation of electron–hole pairs and augment the production of ROS by providing electron traps. Liu et al. [294] synthesized a new kind of oxygen-deficient Gd-doped ZnO nanobullet (named D-ZnO_x_: Gd) to augment the sonodynamic effect of ZnO for deep-seated cancer elimination. The rich oxygen vacancies not only induced the separation of electron–hole pairs in the nanobullets upon US irradiation but also endowed them with an enhanced ability to absorb O_2_^+^ and H_2_O, which significantly boosted the generation efficiency of ROS. Furthermore, the excellent photothermal conversion ability of D-ZnO_x_: Gd under NIR-II laser irradiation renders it highly suitable for PTT, synergizing with SDT to achieve remarkable efficacy in eradicating cancer cells. It has also been shown that integrating metal-based semiconductor nanomaterials with conducting substrates, such as graphene oxide, reduced graphene oxide, and MXenes, can promote the separation of electron–hole pairs by facilitating interfacial charge transfer, thus enhancing the ROS production yield and antitumor SDT effect [295–297]. In addition to these transition metal oxides and noble metal nanomaterials, nanoscale MOFs and their derivatives have also been utilized as effective sonosensitizers. For example, Liang et al. [298] prepared biodegradable defect-rich NH_2_-MIL-125(Ti) [D-MOF(Ti)] via a facile hydrogen reduction method. The defects produced could decrease the bandgap of D-MOF(Ti), accelerate its charge transfer, and consequently boost the SDT effect. Moreover, the Ti^3+^ generated during the hydrogenation process enabled D-MOF(Ti) to further amplify ROS production through a Fenton-like reaction. Considering these advantages, D-MOF(Ti) exhibited promising potential as a multifunctional and safe sonosensitizer with excellent ROS generation performance for effective cancer therapy. In another study, Pan et al. [299] used ZIF-8 as a template to synthesize a novel MOF-derived N-doped carbon sonosensitizer (PMCS) for antitumor SDT. The distinctive zinc-centered porphyrin-like structure of PMCS, combined with the increased cavitation nuclei sites provided by micro/mesopores and high surface area, bilaterally enhanced ROS generation and the SDT effect under US irradiation, resulting in outstanding tumor suppression efficiency. Moreover, due to the low energy gap between the highest occupied molecular orbital (HOMO) and the lowest unoccupied molecular orbital (LUMO), many metal complexes can be easily activated by ultrasonic waves to produce ROS, which is conducive to the abrogation of deeply buried tumors. Ma et al. [300] strategically prepared three types of metal-porphyrin complexes (ZnTTP, MnTTP, and TiOTTP) by utilizing 4-methylphenylporphyrin (TTP) as a ligand and subsequently encapsulated them with human serum albumin for antitumor SDT. By tuning different metal centers, they found that MnTTP possesses the lowest HOMO–LUMO gap compared to ZnTTP and TiOTTP, which makes it the most easily excited under identical ultrasonic conditions, resulting in the highest ^1^O_2_ generation yield. Furthermore, the presence of human serum albumin conferred aqueous solubility, reduced toxicity, and enhanced tumor targeting capabilities on these porphyrin complexes. Upon exposure to US, the activatable depth of MnTTP could reach up to 11 mm, leading to complete inhibition of deep-seated tumors and achieving a 100% survival rate. In conclusion, these metal-based nanomaterials, which can act as carriers of sonosensitizers or function as sonosensitizers themselves to eliminate cancer cells, provide a broad therapeutic prospect for antitumor SDT. However, most metal-based nanomaterials are difficult to degrade, and their long-term toxicity may cause harm to the human body that is hard to estimate. Moreover, the TME featuring hypoxia and a high GSH concentration is unfavorable for sustaining a prolonged sonodynamic reaction, which may compromise the therapeutic efficacy of SDT. Therefore, how to improve the biosafety of metal-based nanomaterials and overcome adverse reaction conditions to enhance sonodynamic therapeutic efficiency are crucial issues to be considered for the clinical promotion of SDT. In addition, the current mechanism of SDT remains incompletely elucidated, and the effects of ultrasound parameters, such as wavelength, frequency, sound pressure, pulse width, and acoustic impedance, on the therapeutic efficiency of sonosensitizers require further investigation.

### Biocatalytic therapy

With advancements in biology and nanotechnology, some metal-based nanomaterials have emerged as efficient nanocatalysts for biocatalysis, providing a highly effective approach for cancer therapy. The redox ability of metal-based nanomaterials can generate ROS in specific tumor regions to directly induce cell death, and the catalytic reaction products can further serve as essential substrates for subsequent catalytic processes, ensuring sequential catalysis and consequently amplifying the anticancer efficacy [301]. In light of these notable advantages, extensive research has been undertaken to explore the potential of biocatalytic cancer therapy. Currently, catalytic cancer treatment mediated by metal-based nanomaterials is commonly achieved through two main strategies: 1) TME-responsive chemodynamic therapy to directly kill cancer cells; and 2) nanozyme catalysis to enhance the anticancer efficacy.

CDT is a newly developed non-invasive cancer treatment that utilizes the in situ generation of toxic molecules through the Fenton or Fenton-like reactions in the acidic TME between H_2_O_2_ and metal ions to eliminate cancer cells [302]. Due to the significantly higher level of H_2_O_2_ in the TME compared to that in normal tissues, chemical-stimuli-driven CDT can selectively act at the tumor site while avoiding damage to surrounding healthy tissues [303]. Furthermore, in contrast to PTT, PDT, and SDT, CDT can be performed without the requirement for exogenous laser irradiation or O_2_, thereby overcoming the challenges posed by limited laser penetration depth and hypoxic TME. At present, a large number of nanomaterials based on metal elements (e.g., Fe, Cu, Mn, V, Co, Mo, W, and Ce) have been reported as CDT reagents [304]. Liu et al. [305] prepared Fe_2_P nanorods coated with biocompatible PTMP-PMAA for enhanced CDT. Fe_2_P nanorods could undergo rapid ionization into Fe^2+^ under acidic conditions, subsequently initiating the Fenton reaction to generate hydroxyl radicals (^·^OH) and thereby enabling specific cancer therapy. Additionally, with the aid of the photothermal effect and external US, the efficiency of Fe^2+^-mediated CDT could be considerably improved, ultimately leading to efficient tumor ablation. However, the Fe-mediated Fenton reaction requires an acidic environment with a narrow and optimal pH range of 3–4. The weakly acidic pH of the TME (pH = 6.5–6.9) falls short of meeting this requirement, thereby compromising the efficacy of Fe-based chemodynamic cancer therapy. In contrast, Cu-based nanocatalysts can catalyze H_2_O_2_ to generate more ^·^OH because their catalytic activity is much higher than that of Fe-based nanocatalysts in the weakly acidic TME [306]. Based on this, Liu et al. [307] strategically synthesized a Cu_3_P nanocatalyst for anticancer CDT. The acidic microenvironment facilitated Cu_3_P nanocatalysts in undergoing Fenton-like reactions to produce ^·^OH. Furthermore, under NIR-II light irradiation, the Fenton-like reaction rate was significantly enhanced as the temperature at the tumor site increased. Moreover, the reaction product Cu^2+^ could be reduced by intracellular GSH to Cu^+^, which could act as a reactant for subsequent catalytic reactions, thereby enhancing the anticancer effect. Mn-based catalysts offer many advantages for cancer treatment. In addition to exhibiting optimal CDT performance to generate toxic ^·^OH under weakly acidic conditions through Fenton-like catalytic processes, they can also effectively deplete GSH in the TME via simple redox reactions to enhance the CDT effect. Duan et al. [308] reported a gallic acid-ferrous (GA-Fe) nanodot-loaded hollow mesoporous MnO_2_ nanoparticle (HMDN), in which the pores were sealed with PEI and subsequently coated with PEG for chemodynamic cancer treatment. After entering the cancer cell with the help of PEG and PEI, the HMDN underwent intracellular degradation by overexpressed GSH, resulting in the depletion of reductive GSH and the release of Mn^2+^, Fe^2+^, and GA. Mn^2+^ and Fe^2+^ could react with the innate H_2_O_2_ to generate toxic ^·^OH, and the resulting high-valence metal ions could be further reduced to lower valence states by reductive GA, thereby amplifying the Fenton and Fenton-like reactions for anticancer CDT.

In addition to the commonly employed Fe-, Cu-, and Mn-based nanocatalysts, there have also been reports of some nanomaterials containing other metal elements for anticancer CDT, such as V, Co, Mo, W, and Ce. For example, Sun et al. [309] successfully developed perovskite-type MnVO_3_ nanomaterials with synergistic chemodynamic and sonodynamic activities for cancer treatment. Both V^5+^ and Mn^2+^ in MnVO_3_ nanomaterials could initiate Fenton-like catalytic processes in cancer cells, facilitating the conversion of H_2_O_2_ into noxious ^·^OH for CDT. Furthermore, due to the narrow bandgap and the abundance of electron traps, MnVO_3_ nanomaterials exhibited exceptional sonodynamic performance in generating ^1^O_2_ under US irradiation for SDT. The release of V^5+^ in the weakly acidic TME endowed MnVO_3_ nanomaterials with the capability to consume GSH, further enhancing the anticancer efficiency of V^5+^/Mn^2+^-catalyzed CDT and SDT. Co-based nanomaterials have also been used for effective cancer CDT. Zhu et al. [310] synthesized a series of biodegradable BSA-modified sulfur-deficient CoS_x_ QDs for enhanced CDT against cancer. As the sulfur vacancy density of CoS_x_ QDs increased, the Fenton-like catalytic effect improved, whereas the photothermal heating effect decreased. As a result, the 1:2 feed molar ratio of Co^2+^ to S^2–^ resulted in the excellent synergistic chemodynamic and photothermal properties of CoS_x_ QDs, enabling them to catalyze the conversion of H_2_O_2_ to sufficient ^·^OH for effective cancer eradication through heat-accelerated Fenton-like reactions. This study demonstrated that sulfur defect engineering represents a viable strategy for enhancing the anticancer performance of Co-based CDT reagents. Furthermore, Li et al. [311] reported that Ce^4+^ could simultaneously function as both a Fenton-like catalyst and a GSH-consuming agent, illuminating the potential of doping transition metal oxides with Ce as a feasible approach for constructing cancer CDT nanoplatforms. Although these metal-based nanomaterials with chemodynamic properties that catalyze the conversion of H_2_O_2_ to ^·^OH provide a promising avenue for specific cancer therapy, their limitations, such as insufficient reactants (e.g., H_2_O_2_ or metal ion concentrations), unsuitable reaction conditions (e.g., pH or temperature), and interference from reducing substances in the TME, may compromise the therapeutic efficacy of anticancer CDT. Therefore, designing multifunctional metal-based nanocatalysts to overcome these difficulties remains a crucial concern for achieving effective cancer eradication in the future.

Nanozymes, which are nano-sized artificial materials with enzyme-mimicking catalytic activities, are very popular in cancer treatment. Nanozymes can ensure high catalytic efficiency while surmounting the inherent limitations of natural enzymes, such as poor stability and high production costs. Generally, the nanozymes reported to have anticancer properties are predominantly fabricated using metal-based nanomaterials, such as metal nanomaterials [312], metal compound nanomaterials [313], MOFs [314], and metal-doped COFs [315]. These nanoenzymes used in cancer therapy typically possess the ability to mimic the activities of oxidoreductases, including POD, oxidase (OXD), catalase (CAT), and superoxide dismutase (SOD). Specifically, POD-like nanozymes can degrade H_2_O_2_ to generate toxic ^·^OH for killing cancer cells [316]. OXD-like nanozymes employ O_2_ as a substrate to generate O2^·−^ and H_2_O_2_ [317]. CAT-like activity can catalyze H_2_O_2_ into O_2_ to alleviate the anoxic TME, enhancing the therapeutic effect of O_2_-mediated treatments, such as PDT, SDT, and even immunotherapy [318]. SOD-like activity can facilitate the conversion of O2^·−^ into H_2_O_2_ and O_2_ for further catalytic reactions [319]. However, due to the limitations of hypoxic conditions and substrate self-consumption in the TME, achieving cancer elimination solely through a single catalytic pathway may present challenges. Intriguingly, metal-based nanomaterials with multienzymatic activities hold great potential in both the self-supply of enzyme substrates and the abundant production of ROS for effective cancer therapy. For example, Liu et al. [320] designed AuCuPt-protoporphyrin IX nanozymes that could trigger cascade enzymatic reactions through multienzymatic activities to treat self-adaptive cancers. These cascade multienzyme-mimicking activities involve POD-like, CAT-like, SOD-like, GOD-like, and glutathione peroxidase (GPx)-like activities, which contribute to the cyclic regeneration of H_2_O_2_ and O_2_, and the depletion of GSH and glucose, consequently inducing excessive ^·^OH generation and enhancing sonodynamic therapeutic outcomes. In addition to exhibiting remarkable efficacy in cancer eradication through catalytic or cascade catalytic reactions, metal-based nanozymes can concurrently safeguard normal cells against the detrimental effects of ROS and H_2_O_2_ through CAT- or SOD-like activities. Wang et al. [321] synthesized FePOs nanozymes with trienzyme-like catalytic activities. In the acidic TME, FePOs nanozymes exhibited POD-like activity to kill cancer cells, while they displayed SOD- and CAT-like activities in neutral or slightly alkaline healthy tissues to prevent damage caused by exogenously injected or endogenously generated H_2_O_2_. Consequently, by constructing pH-sensitive nanozymes with multienzymatic activities, highly efficient therapeutic effects with minimal adverse effects can be achieved. However, conventional nanozymes have only a few active sites and low atomic utilization, which may limit their catalytic performance. Therefore, increasing the atomic utilization of nanozymatic catalytic active centers has the potential to elevate the therapeutic efficacy of catalytic therapy.

With the rapid development of atomic nanotechnology, single-atom nanozymes (SAzymes) are emerging as more potent biocatalysts compared to conventional nanozymes due to their unique advantages, such as well-defined structures, adjustable coordination microenvironments, and maximum metal-atom utilization [322]. For example, modulating the number of coordinated N atoms surrounding the metal active centers, such as by introducing axial N-coordination, can induce an asymmetric electron distribution within SAzymes and augment their catalytic activities [323]. Inspired by this, Liu et al. [324] prepared five-coordinated Ir-N_5_ SAzymes with multi-enzyme mimicking activities. Ir-N_5_ could not only generate abundant ROS through POD- and OXD-like activities but also produce H_2_O_2_ and O_2_ by imitating the activities of CAT and nicotinamide adenine dinucleotide oxidase (NOX), thereby ensuring the self-supply of substrates in the cycle-like catalytic system and enhancing redox damage to cancer cells. Furthermore, the disrupted NADH/NAD^+^ balance caused by Ir-N_5_ with NOX-like activity, in conjunction with cerulenin’s inhibitory effect on fatty acid oxidation, resulted in perturbations in cancer cell energy metabolism homeostasis. This SAzyme, with the ability to simultaneously induce intensive oxidative stress and block energy metabolism, offers a novel horizon for cancer catalytic therapy. In addition, the substitution of weakly electronegative atoms for coordinating N in the metal-N_4_ moieties can improve the adsorption energy of the intermediate, enhancing the enzymatic catalytic performance of SAzymes. Based on this, Liu et al. [325] developed Co-PN_3_ SAzymes loaded with cholesterol oxidase (CHO) to enhance the efficacy of catalytic therapy. Compared to Co-N_4_ SAzymes, Co-PN_3_ SAzymes exhibited enhanced OXD- and CAT-like activities, which upregulated ROS generation within the TME and alleviated tumor hypoxia. The elevated O_2_ level could further activate CHO to consume cholesterol, leading to H_2_O_2_ generation and lipid raft destruction. This process not only increased oxidative stress in cancer cells by providing abundant redox substrates for Co-PN_3_ SAzyme-mediated catalytic reactions but also effectively impeded lamellipodia formation, consequently inhibiting cancer growth, invasion, and metastasis.

Overall, the rapid development of nanozymes has opened new avenues for efficient cancer treatment. However, the multiple enzyme-mimicking abilities of metal-based nanozymes have the potential to engender a competitive dynamic, which may compromise their therapeutic efficacy in treating malignancies. In addition, nanozymes are often difficult to dissolve and degrade, potentially causing long-term adverse effects. Therefore, the identification of fundamental principles directing the design of nanozymes and the acquisition of their biosafety data are crucial for future research in anticancer therapeutics.

### IIT

Metal ions exist widely in living organisms and play an integral role in maintaining the normal physiological activities of biological systems, such as material transportation, cellular communication, and metabolic regulation [326]. Aberrant increases or decreases in intracellular ion levels and abnormal distributions of ions can disturb these physiological processes, leading to irreversible cellular damage and death. Therefore, metal ions can be used to treat a wide range of cancers without inducing drug resistance [23]. However, cancer cells possess a robust self-regulatory capacity to rectify aberrant ion distribution and maintain their inherent physiological homeostasis, posing a significant challenge for effective IIT. Fortunately, the emergence of metal-based nanoagents has provided a promising opportunity for ion accumulation in cancer cells. Metal-based nanomaterials can be internalized into cancer cells via receptor-mediated endocytosis or macropinocytosis and subsequently liberate excessive amounts of metal ions beyond the self-regulatory threshold upon specific stimuli, thereby initiating ion-induced anticancer treatment [196]. As mentioned earlier, many metal ions (e.g., Fe^2+^, Cu^2+^, and Mn^2+^) possess redox capacity and can mediate biocatalysis, causing oxidative damage to cancer cells. Metal ions can also kill cancer cells through other pathways, including by interfering with osmolality (e.g., Na^+^, K^+^, and Ba^2+^), inhibiting signal transduction (e.g., Ca^2+^ and Zn^2+^), and damaging targeted DNA (e.g., Pt^2+^ and Ag^+^).

Asymmetric ionic gradients inside and outside animal cells play an essential role in maintaining normal cellular morphology, structure, and physiological functions [327]. If there is a substantial increase in intracellular Na^+^ or K^+^ concentration, it will result in a corresponding elevation of intracellular osmotic pressure, finally leading to cellular edema, rupture, and death [328, 329]. Based on this, our group developed novel phospholipid-modified Na_2_S_2_O_8_ (PNSO) nanomaterials to deliver Na^+^ and generate ROS for cancer treatment [330]. After entering cancer cells through endocytosis, PNSO nanomaterials could undergo degradation and subsequently release Na^+^ and S_2_O_8_^2−^. The excessive accumulation of intracellular Na^+^ disrupted the osmotic pressure equilibrium between the interior and exterior of cancer cells, ultimately leading to cell swelling and lysis. Furthermore, the conversion of S_2_O_8_^2−^ into highly toxic ^·^SO_4_^−^ and ^·^OH could result in severe oxidative damage to cancer cells. PNSO nanomaterials could also elicit pyroptosis and trigger systemic anticancer immunity, effectively suppressing cancer metastasis and preventing relapse. In addition to serving as ion transport agents, metal-based nanomaterials can act as ion channel blockers to disrupt the osmotic balance of cancer cells. In light of the tendency of K^+^ channels to be overexpressed in a wide range of cancer types and exhibit high variability, they have emerged as promising therapeutic targets for anticancer interventions [331]. Based on this, Zhang et al. [332] prepared a type of Ba^2+^-based nanoagent (GL-BaO_2_ nanomaterials) as a K^+^ channel inhibitor to kill cancer cells. In their work, the surface of BaO_2_ nanomaterials was coated with the chelator *N,N*-bis(carboxymethyl)-_L_-glutamic acid tetrasodium salt (GLDA) to mitigate its adverse effects in vivo by effectively binding free Ba^2+^ in healthy tissues. However, upon X-ray irradiation at the tumor site, ^·^OH generated by BaO_2_ nanomaterials could disrupt the structure of GLDA and release abundant Ba^2+^ to specifically bind to K^+^ channels, leading to the blockage of K^+^ efflux, osmotic imbalance, and finally cell death. This Ba^2+^-based external stimuli-responsive therapeutic strategy provides a promising approach to achieve effective and safe cancer therapy.

Cell signaling is the basis of various biological effects. Similar to normal cells, cancer cells are regulated by various signals. As crucial intracellular messengers, Ca^2+^ and Zn^2+^ have received considerable attention in the realm of anticancer IIT. Ca^2+^ signaling plays a pivotal role in the regulation of cancer cell proliferation, invasion, and metastasis [333]. Zn^2+^, as a second messenger, can not only affect the processes of cancer differentiation, development, and apoptosis, but also engage in cross-talk with Ca^2+^ signaling [334–336]. An excess of Ca^2+^ or Zn^2+^ in cancer cells can potentiate mitochondrial respiration, induce oxidative stress, and result in irreversible cellular demise. For example, Zhang et al. [337] used CaO_2_ nanomaterials to induce “calcium overload” in cancer cells and presented a novel therapeutic approach based on bioactive Ca^2+^ for cancer treatment. Under acidic conditions, CaO_2_ nanomaterials undergo decomposition to generate H_2_O_2_ and Ca^2+^. Benefiting from the downregulated expression of CAT in cancer cells, H_2_O_2_ can accumulate in these cells and induce sustained oxidative stress. This phenomenon disrupts the function of Ca^2+^-related channels, resulting in intracellular calcium overload, ultimately leading to dysfunction in Ca^2+^ signal transduction and cell death. Although many Ca- or Zn-based nanomaterials, such as ZnO, CaO_2_, and CaCO_3_, can kill cancer cells by disturbing ion homeostasis, their low stability tends to result in early release of Ca^2+^ and Zn^2+^, which is detrimental to effective anticancer IIT. Therefore, improving the intrinsic properties of Ca- or Zn-based nanomaterials to achieve satisfactory ionic therapeutic effects with reduced side effects remains a key challenge to overcome in the future.

In addition to interfering with osmotic pressure and cell signal transduction, some metal ions exert anticancer effects by inducing DNA damage. As a typical class of chemotherapy drugs, Pt-containing agents such as cisplatin can bind to DNA and distort its structure, causing cell cycle arrest and cell apoptosis [338]. However, they also have limitations such as low tumor targetability, poor bioavailability, and a high tendency to induce tumor resistance, which are primary contributors to the failure of chemotherapy [339]. Designing Pt-containing anticancer drugs as Pt(IV)-based prodrugs is a promising strategy for improving their therapeutic effects. Pt(IV) prodrugs are inactive in normal tissues, but can be converted to cytotoxic Pt(II) via a simple redox reaction in the TME, which contains high levels of reducing substances. This could enhance the tumor-targeting ability of Pt-based drugs while concurrently mitigating systemic side effects [340]. Furthermore, the consumption of GSH by Pt(IV) in the TME can reduce the possibility of a reaction between activated Pt(II) and GSH, thus overcoming the drug resistance of Pt-based drugs [341]. Based on these advantages, Bi et al. [342] reported an intelligent nanoplatform composed of Pt(IV) prodrug-loaded MnO_2_ nanosheets conjugated to Au_25_ nanoclusters. Upon uptake by cancer cells, MnO_2_ nanosheets were rapidly degraded by intracellular GSH, releasing Au_25_ nanoclusters and Pt(IV) prodrugs. Pt(IV) prodrugs could then be activated into highly poisonous Pt(II) for targeted anticancer chemotherapy while simultaneously achieving dual depletion of GSH. The reduction in GSH could further contribute to an enhanced photodynamic effect triggered by Au_25_ nanoclusters under NIR laser irradiation. The Pt(IV) prodrug-based multifunctional nanoplatform provides a safe and effective strategy for anticancer applications.

Despite significant progress in metal-ion-mediated cancer therapy, IIT alone appears to be insufficient for completely inhibiting tumor growth and metastasis. This may be associated with the circulation time and disassembly efficiency of metal-based nanomaterials, as well as the metabolic activity of cancer cells. Therefore, it is necessary to explore the synergistic anticancer effects of diverse metal ions or combine IIT with other therapeutic modalities to enhance its therapeutic efficacy.

### Immunotherapy

Cancer immunotherapy is a promising therapeutic modality that restores or upregulates the immune system to combat cancer cells. Compared to conventional treatments, immunotherapy has the potential to lead to enduring anticancer responses and reduce the risk of metastasis and relapse caused by immunosuppression and immune evasion [343]. In this regard, metal-based nanomaterials have been proven to be valuable tools for enhancing host anticancer immune function. In addition to serving as carriers to deliver immunomodulatory substances to specific cancer regions, as discussed earlier, metal-based nanomaterials can also effectively exert cancer immunotherapy effects through two aspects: (1) inducing ICD to release tumor-associated antigens (TAAs); and (2) acting as potent adjuvants to enhance the immune response.

ICD denotes a distinct mode of cell death that can initiate a cascade of damage-associated molecular pattern (DAMP) events, including the translocation of calreticulin (CRT) from the endoplasmic reticulum (ER) to the cell surface and the release of adenosine triphosphate (ATP) and high mobility group box 1 (HMGB1) from dying cancer cells. These processes facilitate the presentation of TAAs by APCs, eliciting the proliferation and activation of T cells to infiltrate the TME and mount an effective anticancer immune response, potentially leading to complete cancer eradication [344, 345]. ROS production and ER stress are the primary pathways through which metal-based nanomaterials exert anticancer activities as ICD inducers. Metal-based drugs containing Pt, Ru, Ir, Cu, and Au have been reported to induce ICD responses in cancer cells by increasing ROS levels and activating three typical DAMP signals (CRT, ATP, and HMGB1) [346]. In addition, it has been confirmed that some metal-based nanomaterials with photodynamic, sonodynamic, chemodynamic, and enzyme catalytic activities can improve TAA presentation and stimulate sufficient activation of anticancer immune responses by generating a substantial amount of ROS to trigger CRT exposure and release cancer cell fragments [347–349]. For example, Li et al. [350] prepared a multifunctional nanoagent composed of Fe-porphyrin-based MOFs doped with Pt nanomaterials (termed as FTP), which was then coated with red blood cell membranes to prolong its circulation time and enhance its tumor accumulation capability. FTP could catalyze the generation of O_2_ and the consumption of GSH through CAT-like and GPx-like activities, enhancing the efficiency of PDT and CDT to induce radical storms in cancer cells. The generated ROS effectively evoked severe ICD in cancer cells by enhancing tumor oxidative stress and releasing TAAs and DAMPs, which then initiated systemic immune responses in combination with immune checkpoint blockade antibodies through eliciting inflammatory responses, promoting dendritic cell maturation, and activating CD8^+^ T lymphocytes to achieve long-term control of the growth of primary and distant tumors [350]. Tan et al. [351] designed TME-responsive metal nanoagents with sonodynamic TiO_2_ as the core and acid-sensitive CaP as the shell. When exposed to a pathologically acidic TME, the CaP shell was etched, releasing Ca^2+^ ions and inner TiO_2_. This process not only triggered Ca^2+^ overloading to destroy mitochondrial function but also activated SDT-mediated ROS generation at the specific cancer site, synergistically amplifying cancer cell apoptosis and subsequently enhancing ICD-related anticancer immunotherapy. Interestingly, metal-based nanomaterials can also exert significant ICD-inducing effects by converting laser or alternating magnetic fields into thermal energy to ablate cancer cells and increase the immunogenicity of dying cells. For example, Au nanomaterials with NIR-II photothermal performance evoked a more homogeneous liberation and dispersion of DAMPs in the depths of cancers, leading to significant ICD and stimulation of both innate and adaptive immunity, which could achieve effective cancer regression and metastasis prevention [352]. However, the efficacy of PTT-driven ICD is often compromised by limited tissue penetration depth and poor targeted killing capability. Magnetic hyperthermia therapy mediated by magnetic nanomaterials exhibits inherent advantages in addressing these challenges, which can provoke specific immunotherapy against deep-seated cancer [353]. Pan et al. [354] developed superparamagnetic CoFe_2_O_4_@MnFe_2_O_4_ nanomaterials as ICD inducers for the treatment of primary and distant metastatic cancers. Although metal-based nanomaterials have been demonstrated to induce potent ICD for cancer and metastasis eradication, the precise mechanism by which this is achieved remains elusive. Further exploration is necessary to understand how metal-based nanomaterials provoke ER stress and disrupt protein synthesis. Moreover, the biosafety of metal-based nanomaterials remains a key issue in cancer immunotherapy that requires systemic administration. Therefore, thoroughly investigating the impacts of metal-based nanomaterials on the intricate immune system and developing metal-based nanomaterials with low biotoxicity and strong ICD-inducing effects may have greater significance in practical biological anticancer applications.

Although ICD can provide TAAs, the satisfactory efficacy of anticancer immunotherapy generally requires additional immune adjuvants that can significantly enhance immune activation. In this regard, it has been confirmed that metal ions (e.g., Al^3+^, Mn^2+^, Zn^2+^, Co^2+^, Ca^2+^, Mg^2+^) play a vital role in the regulation of immune processes. Al-based adjuvants such as alum have been widely employed in clinical settings because of their ability to activate the NLRP3 inflammasome, stimulate PI3K-Syk kinase signal transduction, and trigger strong antigen-specific humoral and cellular immunity [355]. Inspired by these developments, combining the advantages of nanotechnology and metal ions to construct novel adjuvants has sparked significant interest. It has been reported that Mn^2+^ can promote comprehensive activation of the cGAS-STING pathway by increasing cGAMP production, enhancing the detection sensitivity of cytosolic dsDNA by cGAS, and reinforcing the binding affinity between cGAMP and STING. Activation of this pathway then triggers the secretion of type I interferon and inflammatory cytokines, eliciting immune responses to fight against cancer [356–358]. Based on this, Hou et al. [358] developed phospholipid-modified DOX-loaded amorphous porous manganese phosphate nanomaterials, which exhibit dual responsiveness to phospholipase and pH. Upon release in the TME, DOX could elicit DNA damage, leading to the activation of cGAS. Additionally, elevated intracellular Mn^2+^ levels intensify STING activity, thereby facilitating the initiation of the cGAS-STING pathway. These processes not only resulted in the release of TNF-α and IL-6 but also promoted dendritic cell maturation and strengthened the anticancer immune responses mediated by cytotoxic T lymphocytes or natural killer (NK) cells. Similar to Mn^2+^, Zn^2+^ and Co^2+^ can amplify STING-dependent immune responses [359, 360]. Du et al. [360] further confirmed that Zn^2+^ bolsters the enzyme activity of intracellular cGAS by facilitating the phase separation of cGAS-DNA. Inspired by these findings, Zhang et al. [361] constructed Zn-doped LDH (Zn-LDH) as a nanoadjuvant to potentiate cancer immunotherapy against solid tumors. After internalization into the cancer cell, Zn-LDH effectively elevated the pH level of acidic lysosomes, thereby spoiling the endosome/lysosome and inhibiting autophagy while evoking mitochondrial damage and DNA release. On this basis, Zn^2+^ released in cancer cells activated and intensified the cGAS-STING signal, strikingly inducing ICD and promoting TAA presentation. Moreover, Zn-LDH further amplified immune activation by downregulating the expression of PD-L1 and CD47. Consequently, by reversing cancer immunosuppression and eliciting antigen-specific anticancer immunity, the Zn-LDH nanoadjuvant successfully inhibited cancer without the need for additional immunomodulators. In addition to activating the cGAS-STING pathway, metal ions such as Mg^2+^ and Ca^2+^ can enhance anticancer immune responses by directly improving the effector functions of immune cells. For instance, Mg^2+^ participates in various immune signaling pathways and plays an important role in the activation of NK, T, and B cells [362, 363]. Ca^2+^, as a second messenger, can be involved in complex immune processes, such as inducing cytotoxic T lymphocytes and NK cells to release perforin and granzymes and express high levels of death receptors, facilitating T cell activation and proliferation, promoting B cell development, and activating the NLRP3 inflammasome [364]. A study has demonstrated that the use of CaCO_3_ nanomaterials can dramatically enhance intracellular Ca^2+^ levels, resulting in an elevated ratio and number of dendritic cells and macrophages in the spleen [365]. CaP nanomaterials have been reported to exhibit strong adjuvant performance in eliciting both cellular and humoral immune responses [24]. However, the development of metal-based nanoadjuvants for cancer immunotherapy is still in its nascent stages, and further systematic research is needed for nanoadjuvants based on other nutrient metal ions, such as K^+^ and Na^+^. In addition, whether other metal ions besides Mn^2+^, Zn^2+^, and Co^2+^ can enhance STING-dependent immune responses has not been thoroughly investigated.

### Multimodal therapy

The integration of multiple therapeutic modalities into a single nanoplatform is anticipated to surmount the limitations of individual therapies, offering potential for effective cancer treatment and the prevention of cancer metastasis and recurrence [366]. Among bimodal therapeutic modalities, the combination of PTT and chemotherapy emerges as a promising strategy due to the localized hyperthermia induced by PTT, which enhances the uptake of nanocarriers by cancer cells and promotes intracellular drug release, thus augmenting the anticancer efficacy of chemotherapeutic agents [164]. Qiang et al. [367] incorporated cisplatin prodrugs [Cispt(IV)] and CuS nanomaterials into a PEGylated Fe(III)-based MOF to develop an innovative nanoplatform (M-Pt/PEG-CuS) for tumor-specific synergistic PTT/chemotherapy. In vitro studies confirmed that the synthesized products exhibited a temperature elevation of up to 45°C under 1064 nm laser irradiation, leading to cancer cell death via thermal ablation. Additionally, the generated heat enhanced the uptake of the prepared products and facilitated the release of Cispt(IV), resulting in a significant decrease in cancer cell viability. In vivo studies confirmed that the M-Pt/PEG-CuS-mediated PTT/chemotherapy synergistic effect exhibited the most potent induction of cell apoptosis and the highest tumor suppressive efficacy, surpassing those of free chemotherapeutic drugs or PTT alone. Notably, photothermal/chemo combinational therapy induced by metal-based nanomaterials has demonstrated high efficacy in simultaneously eliminating primary tumors and distant metastases. Sun et al. [368] prepared biomimetic nanosystems (CDAuNs) comprising gold nanocages (AuNs) coated with 4T1 cancer cell membranes and loaded with DOX for PTT-enhanced chemotherapy. They reported that CDAuNs could effectively localize to the source cancer cells, including both primary tumors and metastases, owing to the homotypic targeting of cancer cell membranes. The increased temperature induced by AuNs upon NIR laser irradiation accelerated the release of DOX and facilitated the intracellular accumulation of CDAuNs by enhancing the permeability of tumor cell membranes. The in vivo results revealed that CDAuNs achieved a striking reduction in 4T1 tumor volume of up to 98.9% and effectively suppressed lung metastatic nodules by 98.5% through the synergistic killing effect of NIR laser-induced hyperthermia and high concentrations of DOX. Therefore, CDAuN-induced synergistic PTT/chemotherapy could serve as a novel and effective strategy for inhibiting tumor growth and metastasis.

Similar to PTT-enhanced chemotherapy, photothermal heating can boost the uptake of PSs-loaded metal-based nanomaterials by tumor cells, which contributes to the intracellular accumulation of PSs and the generation of large amounts of ROS for enhanced antitumor PDT efficacy [369]. For instance, Liu et al. [366] designed a tumor-targeted nanosystem (named MoSe_2_@ICG-PDA-HA) consisting of ICG-loaded MoSe_2_ nanomaterials functionalized with polydopamine (PDA) and HA for bimodal PTT/PDT therapy. The nanosystem demonstrated high efficiency in releasing ICG in the weakly acidic TME due to the pH-sensitive PDA coating on the surface of MoSe_2_ nanomaterials. Furthermore, the photothermal effect mediated by MoSe_2_ nanomaterials could further enhance the release of ICG by weakening its physical adsorption, thereby amplifying the efficacy of PDT. The results of in vivo experiments confirmed that upon NIR irradiation, the synergistic PTT/PDT mediated by MoSe_2_@ICG-PDA-HA exhibited superior efficacy in suppressing tumor growth and inhibiting metastasis compared to MoSe_2_ nanomaterials or ICG alone.

In addition to the two synergistic therapeutic strategies mentioned above, other bimodal therapeutic modalities mediated by metal-based nanomaterials can also offer promising clinical advantages in terms of cancer elimination and prolonged survival. For example, the high temperature induced by MoS_2_ nanosheets upon NIR laser irradiation can significantly enhance the efficiency of the Fe^3+^-mediated Fenton reaction, enabling photothermal-enhanced CDT/PTT to completely eradicate cancer [370]. Moreover, chemotherapeutic prodrugs, such as oxaliplatin [Oxa(IV)], can be reduced to toxic Oxa(II) by GSH in cancer cells, and GSH depletion facilitates the efficient intracellular accumulation of ^·^OH generated through Mn^2+^-mediated Fenton-like reactions. Synchronous CDT/chemotherapy using metal-based nanomaterials has dramatically enhanced antitumor effects compared to their applications [371]. In addition, radiotherapy can help to remodel the tumor immune microenvironment, alleviate immunosuppression, and trigger T-lymphocyte-mediated immune responses, leading to effective tumor suppression [372]. Wang et al. [373] developed siRNA-modified Au/MnO_2_ nanomaterials for enhanced tumor radio-immunotherapy. MnO_2_-catalysed O_2_ generation could alleviate tumor hypoxia and enhance Au-mediated radiosensitizing effects, leading to tumor cell death and the subsequent induction of ICD. Meanwhile, siRNA could downregulate PD-L1 expression and alleviate radiotherapy-induced tumor immunosuppression, thereby enhancing T-cell antitumor immune responses. Synergistic radio-immunotherapy is an effective method for inhibiting tumor growth.

The use of metal-based nanomaterials in trimodal therapy is expected to result in optimal anticancer outcomes with a reduced administered dose. The combination of chemotherapy, PTT, and PDT holds great promise because of their synergistic effects. As discussed earlier, the photothermal effect mediated by metal-based nanomaterials can boost the anticancer efficacy of both chemotherapy and PDT. In addition, ROS generation during PDT may improve intracellular drug delivery by enhancing the endosomal escape effect of chemotherapeutic agents. The synergistic use of PTT, PDT, and CDT has also demonstrated remarkable efficacy in cancer eradication. The heat generated by PTT enhances the PDT effect by increasing the cellular uptake of PSs and alleviating tumor hypoxia. Additionally, PTT-enhanced CDT can be achieved by improving the efficiency of Fenton or Fenton-like reactions, and the O_2_ generated from these reactions can further amplify the PDT efficacy. Our group synthesized copper ferrite nanospheres (named CFNs) for synergistic PTT/PDT/CDT [374]. The presence of Fe^3+^ and Cu^2+^ ions in CFNs modulated the TME to improve the efficiency of PDT through catalytic reactions that generate O_2_ and deplete GSH. The Fenton and Fenton-like reactions mediated by these metal ions were markedly enhanced under 650 nm laser irradiation, which effectively suppressed tumor growth. Furthermore, CFNs were found to be capable of raising the temperature high enough to ablate tumors upon irradiation with an 808 nm laser. The combination of CFNs with two wavelengths of light demonstrated a potent synergistic anticancer effect in PTT, PDT, and CDT, leading to complete tumor eradication in vivo. In addition, Meng et al. [375] strategically designed sulfur-deficient Bi_2_S_3−x_-Au@HA heterostructure nanocomposites for synergistic anticancer SDT/PDT/PTT. The plasmonic Au nanomaterials evoked LSPR, intensively enhancing the surrounding electric field and endowing Bi_2_S_3−x_-Au@HA with strong NIR-II absorption ability and excellent photothermal heating performance. Furthermore, upon NIR-II laser and US irradiation, the presence of sulfur vacancies and Schottky heterojunction synergistically promoted charge separation and inhibited electron–hole recombination, significantly increasing the ROS yield. As a result, defective Bi_2_S_3−x_-Au@HA heterostructures can achieve effective tumor eradication by inducing the synergistic effects of SDT, PDT, and PTT. In addition, a synergistic approach combining IIT, CDT, and immunotherapy has shown significant antitumor efficacy. Mo et al. [376] synthesized CaO_2_@CUR@ZIF-Cu nanoplatforms for synergistic IIT/CDT/immunotherapy. CaO_2_ and curcumin induced intracellular Ca^2+^ overload, which subsequently promoted apoptosis in tumor cells. The H_2_O_2_ generated from the hydrolysis of CaO_2_ in the TME enhanced the Cu-mediated CDT effect, while apoptosis induced by Ca^2+^ overload and CDT further activated the systemic antitumor immune response, remarkably strengthening the antitumor therapeutic effect.

In short, using the unique physicochemical properties of metal-based nanomaterials to construct multifunctional nanoplatforms can help achieve much higher anticancer efficacy than single cancer therapies. However, metal-based nanomaterial-mediated multimodal therapies are mostly in the laboratory phase and face several challenges before practical clinical application, including dose optimization, patient selection, and regulatory approval. The pharmacokinetic properties of metal-based nanomaterials in vivo significantly influence their concentration and duration in target tissues. To optimize the dosage, it is essential to select an appropriate tumor-bearing mouse model for each metal-based nanomaterial to investigate its pharmacokinetics, ensuring its efficacy while strictly avoiding toxicity to normal tissues. Before clinical trials, patient stratification is needed to predict which patients are most likely to benefit from multifunctional metal-based nanomaterials. Finally, rigorous biosafety and efficacy evaluations through multi-phase clinical trials are required before the introduction of a new multimodal treatment. If positive clinical trial results can be achieved, appropriate regulations need to be followed to submit an application to the regulatory authorities for clinical translation. Therefore, persistent efforts to achieve a harmonious integration of metal-based nanomaterials with exceptional anticancer properties and biosafety will facilitate their extensive application in future clinical cancer treatments.

## Conclusions

In recent years, the development of imaging probes and therapeutic agents based on metal-based nanomaterials has progressed rapidly, providing a powerful tool for early personalized cancer diagnosis and treatment. In terms of efficient cancer diagnosis, metal-based nanomaterials with exceptional X-ray attenuation, magnetic properties, and optical characteristics provide an opportunity to achieve high-sensitivity and high-contrast cancer imaging. From a therapeutic perspective, various types of metal-based nanomaterials with unique properties have been developed to achieve targeted distribution at the tumor site and to surmount the limitations posed by TME. Furthermore, the development of multifunctional metal-based nanomaterials for multimodal synergistic therapies offers an efficient and robust strategy for complete tumor eradication and metastasis prevention.

To date, although the development and utilization of metal-based nanomaterials have achieved satisfactory outcomes in various aspects of cancer diagnosis and treatment, they are mostly in the laboratory research stage. Only a limited number of metal-based cancer nanomedicines have received clinical approval. Specifically, NBTXR3/Hensify® has gained approval for its application in enhancing the efficacy of radiotherapy for soft tissue sarcomas [244]. NanoTherm® is an aminosilane-coated superparamagnetic Fe_3_O_4_ nanomaterial, which assists in enhancing radiotherapy for glioblastoma through intratumoral hyperthermia [377]. In addition, PEGylated gold nanomaterials bound with tumor necrosis factor have successfully progressed to phase I clinical trials for the treatment of solid tumors [378].

Although the aforementioned nanomaterials based on Hf, Fe, and Au have exhibited promising outcomes in clinical translation, metal-based nanomaterials still face several challenges that need to be overcome for their widespread application in clinical settings. The toxicity and biosafety of metal-based nanomaterials are key factors that hinder their clinical translation. The modification of metal-based nanomaterials using highly biocompatible and degradable materials has been shown to effectively reduce their short-term cytotoxicity while optimizing their in vivo clearance. Nevertheless, the long-term cytotoxicity of metal-based nanomaterials necessitates further investigation. In-depth studies are necessary to elucidate the reaction processes and pharmacokinetic properties of metal-based nanomaterials in vivo and to explore the complex mechanisms of their interactions with living organisms. Prioritizing metal elements with low toxicity, such as Au, Fe, and Hf, which have been utilized in approved nanomedicines, for the construction of nanomaterials during the future material design phase will help reduce long-term toxicity and facilitate clinical translation. Furthermore, the adoption of green and environmentally friendly production processes to minimize the emission of hazardous substances during the production process, along with the continuous improvement of nanomaterial purity through techniques such as filtration and centrifugation, can significantly reduce the toxic effects caused by impurities.

Moreover, it should be noted that clinical translation requires metal-based nanomaterials that are physicochemically stable and consistent, as well as being low-cost, reproducible, and storable. Standardization of manufacturing processes is essential to ensure consistent quality of nanomaterials. Currently, the International Organization for Standardization has established standardized regulatory requirements covering various topics, such as physicochemical properties, toxicity testing, and risk assessment. However, some unaddressed needs remain for the standardization of nano-risk review and governance, which may require a long-term process [379]. In addition, the correlation between data from preclinical animal studies and human trials is weak. Therefore, before clinical trials, patients should be screened to determine the specific patient populations and tumor types for which metal-based nanomaterials are most likely to be effective. Thus, to facilitate the clinical translation of metal-based nanomaterials, it is crucial to address several key aspects, including large-scale production, optimization of storage conditions, patient stratification, tumor type screening, and continuous refinement and updating of regulatory requirements.

In summary, metal-based nanomaterials exhibit significant potential for precise cancer diagnosis and treatment. To enhance the clinical applicability of metal-based nanomaterials, it is imperative to conduct further investigations and refine their formulations. It is believed that the development and application of reproducible metal-based nanomaterials with exceptional tumor targeting, biocompatibility, and low cytotoxicity will help to fulfill the urgent clinical demand for efficient and personalized cancer diagnosis and therapy.

## Data Availability

Not applicable.

## Abbreviations

3pRNA: 5′-Triphosphate RNA
5-FU: 5-Fluorouracil
AIPH: 2,2-Azobis[2-(2-imidazolin-2-yl)propane] dihydrochloride
APCs: Antigen-presenting cells
ATP: Adenosine triphosphate
AuNCs: Gold nanoclusters
AuNs: Gold nanocages
Bi-QCS: Biotin-quat188-chitosan
BMSNs: Bi-based mesoporous-silica-coated nanomaterials
BSA: Bovine serum albumin
CAT: Catalase
CDT: Chemodynamic therapy
Ce6: Chlorin e6
cGAS: Cyclic GMP-AMP synthase
CHO: Cholesterol oxidase
COFs: Covalent organic frameworks
CpG-ODN: Cytosine-phosphate-guanine oligodeoxynucleotides
CRT: Calreticulin
CT: Computed tomography
DAMP: Damage-associated molecular pattern
DDSs: Drug delivery systems
DNA: Deoxyribonucleic acid
DOX: Doxorubicin
EPR: Enhanced permeability and retention
ER: Endoplasmic reticulum
FL: Fluorescence
GA-Fe: Gallic acid-ferrous
GLDA: *N,N*-Bis(carboxymethyl)-_L_-glutamic acid tetrasodium salt
GNRs: Gold nanorods
GOD: Glucose oxidase
GPx: Glutathione peroxidase
GSH: Glutathione
HA: Hyaluronic acid
HMDN: Hollow mesoporous MnO_2_ nanoparticle
HMGB1: High mobility group box 1
HOFs: Hydrogen-bonded organic frameworks
HOMO: Highest occupied molecular orbital
ICD: Immunogenic cell death
ICG: Indocyanine green
IIT: Ion interference therapy
IONCs: Iron oxide nanoclusters
Iso4: Cyclic [CphgisoDGRG] peptide
LDHs: Layered double hydroxides
L-EGCG-Mn: L-epigallocatechin gallate complexed Mn^2+^
LSPR: Localized surface plasmon resonance
LUMO: Lowest unoccupied molecular orbital
MOFs: Metal-organic frameworks
MRI: Magnetic resonance imaging
NBs: Nanobundles
NIR: Near-infrared
NIR-I: First near-infrared
NIR-II: Second near-infrared
NK: Natural killer
NLS: Nuclear localization signal
NOX: Nicotinamide adenine dinucleotide oxidase
^1^O_2_: Singlet oxygen
^·^OH: Hydroxyl radical
OXD: Oxidase
PA: Photoacoustic
PAA: Polyacrylic acid
PAI: Photoacoustic imaging
PCE: Photothermal conversion efficiency
PDA: Polydopamine
PD-L1: Programmed death-ligand 1
pDNA: Plasmid deoxyribonucleic acid
PDT: Photodynamic therapy
PEG: Polyethylene glycol
PET: Positron emission tomography
POD: Peroxidase
PSs: Photosensitizers
PTAs: Photothermal agents
PTT: Photothermal therapy
PVP: Polyvinylpyrrolidone
QDs: Quantum dots
RB: Rose Bengal
RENPs: Rare earth-doped nanoparticles
RES: Reticuloendothelial system
ROS: Reactive oxygen species
SAzymes: Single-atom nanozymes
SDT: Sonodynamic therapy
SH-cNGR: Cyclized asparagine-glycine-arginine peptide
siRNA: Small interfering RNA
SOD: Superoxide dismutase
SPECT: Single-photon emission computed tomography
SPIONs: Superparamagnetic iron oxide nanoparticles
STING: Stimulator of interferon genes
TAAs: Tumor-associated antigens
TME: Tumor microenvironment
TTP: 4-Methylphenylporphyrin
UCL: Upconversion luminescence
UCNPs: Upconversion nanoparticles
US: Ultrasound
UV: Ultraviolet
ZIF-8: Zeolitic imidazolate framework-8
